# Seasonal Variation in Essential Oil Composition and Bioactivity of Three *Ocimum* Species from Nepal

**DOI:** 10.3390/molecules30173581

**Published:** 2025-09-01

**Authors:** Prem Narayan Paudel, Prabodh Satyal, William N. Setzer, Suresh Awale, Shiro Watanabe, Juthamart Maneenet, Rakesh Satyal, Ajaya Acharya, Anjila Shrestha, Rajendra Gyawali

**Affiliations:** 1 Department of Chemical Science and Engineering, Kathmandu University, Dhulikhel 45200, Nepal; prempaudel@ku.edu.np; 2 Aromatic Plant Research Center, 230 N 1200 E, Suite 100, Lehi, UT 84043, USA; 3 Department of Chemistry, University of Alabama in Huntsville, Huntsville, AL 35899, USA; 4 Natural Drug Discovery Laboratory, Institute of Natural Medicine, University of Toyama, 2630 Sugitani, Toyama 930-0194, Japan; suresh@inm.u-toyama.ac.jp (S.A.); shirowat@inm.u-toyama.ac.jp (S.W.); juthamart_pp@hotmail.com (J.M.); 5 Analytica Research Center, Kirtipur 44660, Nepal; rsatyal@aromaticplant.org; 6 Department of Pharmacy, Kathmandu University, Dhulikhel 45200, Nepal; ajaya.acharya@ku.edu.np (A.A.); shresthaanjila83@gmail.com (A.S.)

**Keywords:** volatile composition, *Ocimum* spp., seasonal disparity, chiral GC-MS, biological activity, hierarchical cluster analysis

## Abstract

The plants from the *Ocimum* genus, belonging to the Labiatae family, serve as important bioresources of essential oils (EOs) rich in biologically active secondary metabolites, widely used in medicine, food, and cosmetics. This study explored the volatile composition, enantiomeric distribution, and in vitro biological activities of EOs from three *Ocimum* species native to Nepal: *O. tenuiflorum* L., *O. basilicum* L., and *O. americanum* L. EOs were extracted via hydro-distillation and analyzed using gas chromatography–mass spectrometry (GC-MS) for chemical profiling and chiral GC-MS for enantiomeric composition. Hierarchical cluster analysis was performed for major chemotypes. Antioxidant activity was assessed using DPPH and ABTS assays. Antimicrobial efficacy was evaluated using the microbroth dilution method, and cytotoxicity was tested on NIH-3T3 (normal) and MCF-7 (breast cancer) cell lines via the Cell Counting Kit-8 assay. EO yield was highest in *O. tenuiflorum* (1.67 ± 0.13%) during autumn and lowest in *O. americanum* (0.35 ± 0.02%) during winter among all *Ocimum* spp. The major compounds identified in *O. tenuiflorum* were eugenol (32.15–34.95%), *trans*-β-elemene (29.08–32.85%), and β–caryophyllene (19.85–21.64%). In *O. americanum,* the major constituents included camphor (51.33–65.88%), linalool (9.72–9.91%), germacrene D (7.75–1.83%), and β–caryophyllene (6.35–3.97%). For *O. basicilum,* EO was mainly composed of methyl chavicol (62.16–64.42%) and linalool (26.92–27.05%). The oxygenated monoterpenes were a dominant class of terpenes in the EOs except for *O. tenuiflorum* (sesquiterpene hydrocarbon). A hierarchical cluster analysis based on the compositions of EOs revealed at least three different chemotypes in *Ocimum* species. Chiral GC-MS analysis revealed β-caryophyllene and germacrene D as enantiomerically pure, with linalool consistently dominant in its levorotatory form. *O. tenuiflorum* exhibited the strongest antimicrobial activity, particularly against *Candida albicans*, and showed notable anticancer activity against MCF-7 cells (IC_50_ = 23.43 µg/mL), with lower toxicity to normal cells. It also demonstrated the highest antioxidant activity (DPPH IC_50_ = 69.23 ± 0.10 µg/mL; ABTS IC_50_ = 9.05 ± 0.24 µg/mL). The EOs from *Ocimum* species possess significant antioxidant, antimicrobial, and cytotoxic properties, especially *O. tenuiflorum*. These findings support their potential application as natural agents in medicine, food, and cosmetics, warranting further validation.

## 1. Introduction

The *Ocimum* genus, commonly known as basil, encompasses a diverse group of aromatic herbs that are widely recognized for their culinary, medicinal, and ornamental value. This genus comprises over 150 species, commonly found across temperate zones globally [1]. The *Ocimum* genus is prevalent in tropical zones, particularly in Asia, Africa, India, and Nepal [2]. The most well-known species include highly aromatic herbs such as *Ocimum tenuiflorum*, *Ocimum basilicum*, *Ocimum gratissimum*, and *Ocimum americanum*. They are commonly known as holy basil (or Tulsi), sweet basil, African basil (or clove basil), and hoary basil (or American basil), respectively. Many countries in East Asia, Europe, America, and Australia cultivate these species primarily for their essential oil yields [3]. This genus is characterized by its rich phytochemical profile, which contributes to its wide-ranging applications in traditional medicine and modern therapeutics. The essential oils of *Ocimum* species are extensively employed as high-value aromatic compounds in the food industry (as flavorings), perfumery, cosmetics, pharmaceuticals, and spices. The diverse pharmacological actions of *Ocimum* species, such as carminative, antimicrobial, and antioxidant effects, have also made them valuable in traditional medicine for treating conditions like gastric and urinary diseases, insomnia, inflammation, and constipation [4]. Recent studies have highlighted the bioactive compounds present in *Ocimum* species, including essential oils, flavonoids, and phenolic acids, which exhibit a variety of activities such as antimicrobial, anti-inflammatory, and antioxidant effects. The aerial parts of these plants are traditionally used for their antispasmodic, stomach-soothing, and carminative effects [5]. Antiemetic activity has been documented in both *O. basilicum* and *O. gratissimum* [6]. Recent studies have explored the potential of essential oils from *O. sanctum*, *O. gratissimum*, and *O. basilicum*, particularly for their antioxidant and antimicrobial activities [7].

Essential oils are intricate blends of volatile compounds characterized by their strong aromas and hydrophobic properties. They can be obtained using a variety of extraction techniques from the different plant sections. So, essential oils derived from aromatic plants are known for their complex chemical compositions and diverse biological activities. As per the Annotated Checklist of the Flowering Plants of Nepal, a minimum of four species belonging to this genus are recorded in the Nepalese flora [8]. The plant species from the *Ocimum* genus, particularly those native to Nepal, have garnered significant attention due to their traditional uses and potential therapeutic properties. The most commonly studied *Ocimum* species in Nepal include *Ocimum basilicum* (sweet basil), *Ocimum sanctum* (holy basil or tulsi), *Ocimum gratissimum* (clove basil), and *Ocimum americanum*, each distinguished by its unique volatile constituents and corresponding biological effects.

Seasonal variation plays a crucial role in the composition of essential oils, influencing both the quantity and quality of the volatile compounds produced by plants. Environmental factors such as temperature, humidity, and light intensity can significantly affect the biosynthesis of these compounds, leading to fluctuations in their chemical profiles throughout the year [9,10]. Understanding of these variations is essential for optimizing the extraction and application of essential oils in various fields, including aromatherapy, pharmacology, and agriculture.

Furthermore, the increasing interest in natural products as alternatives to synthetic drugs has further propelled research into the potential health benefits of *Ocimum* species. The literature review indicates that, to the best of our knowledge, this is a comprehensive study on the chemical composition, enantiomeric distribution, seasonal variations, biological efficacies, and cytotoxic effects of EOs from plant species found in Nepal or other regions. Therefore, this study aims to investigate the seasonal variation in the volatile constituents of essential oils from three *Ocimum* species growing in Nepal. By employing gas chromatography–mass spectrometry (GC-MS) analysis, we seek to identify and quantify the major volatile compounds in each species across different seasons. Additionally, we will evaluate the biological activities of these essential oils, focusing on their antimicrobial, antifungal, antioxidant, and cytotoxic effects. The findings could have significant implications for their cultivation, conservation, and application in various industries, ultimately contributing to the sustainable use of Nepal’s rich biodiversity.

## 2. Results and Discussion

In the present study, three *Ocimum* species were investigated to determine the effect of harvesting seasons on the yield and quality of essential oils obtained from the aerial parts (leaves and inflorescence).

### 2.1. Variation in the Yield of Essential Oils

The yields of essential oils of all three *Ocimum* species were found in the range of 0.35 ± 0.02 to 1.67 ± 0.13% (*v*/*w*) (Table 1, Table 2 and Table 3). The minimum EO yields (0.35 ± 0.02%) were found in *O. americanum* during winter, while the maximum yields (1.67 ± 0.13%) of EO of *O. tenuiflorum* were during autumn. These *Ocimum* species demonstrated higher essential oil yield in autumn and winter (plants being in full bloom) than in other seasons. This variation in the essential oil yield must be associated with harvesting season, because previous studies have revealed that a number of factors, including seasonal variation, geographic origin, and variations in extraction techniques (such as temperature and extraction time), could contribute to the variations in EO yields [11]. However, EO yields were found to be somewhat better or at least comparable with many previous reports.

### 2.2. Essential Oil Composition

**Figure 1:**
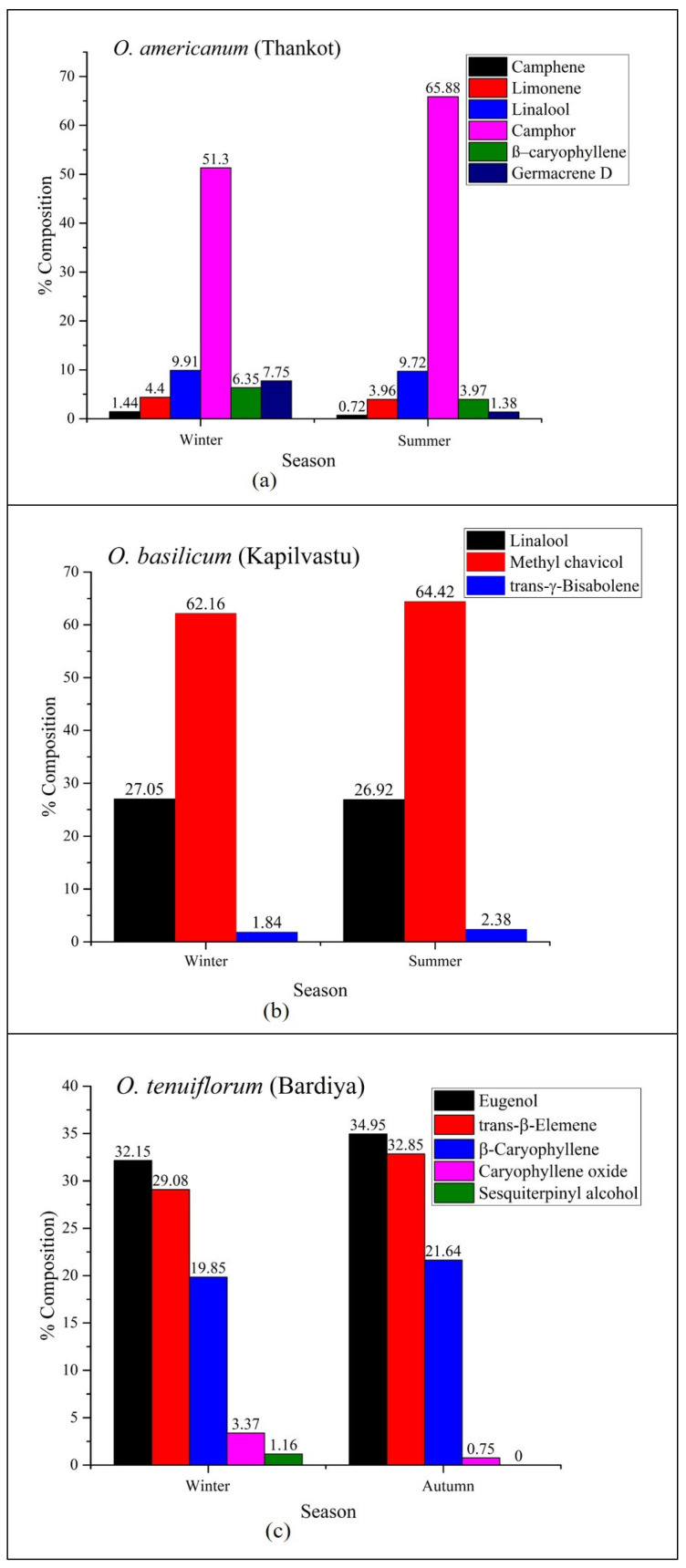
Seasonal variations in the major compounds of essential oil for *Ocimum* species: (**a**) *O. americanum* (Thankot); (**b**) *O. basilicum* (Kapilvastu); and (**c**) *O. tenuiflorum* (Bardiya).

The results of GC-MS analysis of three *Ocimum* species EOs at different seasons are reported in Table 1, Table 2 and Table 3, also shown by Figure 1 and Figures S1–S6, in the SM. The analysis led to the detection and identification of 49 constituents with 98.28% and 53 constituents with 99.71% of total *O. tenuiflorum* essential oil during winter-autumn from Bardiya, 60 constituents with 99.8% and 57 constituents with 99.8% of total *O. americanum* essential oil during winter–summer from Thankot, and 51 compounds with 96.59% and 54 compounds with 99.7% of total *O. basilicum* essential oil during winter–summer from Kapilvastu, respectively. The major constituents (>3%) in the EOs of *O. tenuiflorum* during winter and autumn were found to have eugenol (32.15 to 34.95%), *trans*-β-elemene (29.08 to 32.85%), β–caryophyllene (19.85 to 21.64%), and caryophyllene oxide (3.37 to 0.75%), respectively. The main components of *O. americanum* essential oil collected during winter and summer were camphor (51.33 to 65.88%), linalool (9.91 to 9.72%), germacrene D (7.75 to 1.83%), β–caryophyllene (6.35 to 3.97%), and limonene (4.4 to 3.96%), respectively, while methyl chavicol (62.16 to 64.42%) and linalool (26.92 to 27.05%) were the major constituents in the EOs of *O. basilicum* during winter and summer. Additionally, *Ocimum.* EOs also contained substantial amounts of various minor components, as shown in the respective tables.

Group components were found to be differentiated by a major contribution of oxygenated monoterpenes followed by sesquiterpenes and monoterpene hydrocarbons in both *O. americanum* and *O. basilicum*, whereas sesquiterpene hydrocarbons were the dominating class of terpenes in *O. tenuiflorum*. *O. americanum* and *O. basilicum* essential oils collected during winter and summer consisted of 68.57 to 82.38% and 28.65 to 28.09% oxygenated monoterpenes, respectively. The EOs of *O. americanum* during the summer season recorded higher oxygenated monoterpenes as compared with winter, while the winter season recorded 6.64 to 11.35% higher monoterpene hydrocarbons as compared with summer season, respectively. Relatively higher sesquiterpenes hydrocarbons (62.54%) were recorded for *O. tenuiflorum* EOs during autumn as compared with winter (57.11%), while very low amounts were recorded for *O. americanum* and *O. basilicum* during other seasons. Linalool and camphor were the major oxygenated monoterpenes in *O. americanum* EOs during both the winter and summer seasons, while *O. basilicum* EOs had only linalool as the main oxygenated monoterpene during both seasons.

The variation in the amounts of most of the essential oils investigated in the present study was observed with respect to species and season of harvesting. The fluctuation in the major essential oil components of *O. tenuiflorum* includes eugenol (32.15 to 34.95%) and *trans*-β-elemene (29.08 to 32.85%) from winter to autumn. The major variation in the *O. americanum* essential oil includes camphor (51.33–65.88%), linalool (9.91–9.72%), germacrene D (7.75–1.83%), β–caryophyllene (6.35–3.97%), and limonene (4.4–3.96%) from winter and summer, respectively. Similarly, the major discrepancy observed in the *O. basilicum* EOs was methyl chavicol (62.16–64.42%) and linalool (26.92–27.05%) from winter to summer. The amount of most of the compounds was found to be higher in summer, while eugenol, a valuable antioxidant component, was found to be greater in autumn. This shows that the content of EOs was dependent on the season; however, this did not follow the same trends for all the plants. The variation in the composition of EOs with respect to seasons may be associated with the phenological stage of the plant, while other environmental effects can significantly influence the regulation of EO biosynthesis [9,10]. These findings are largely consistent with numerous earlier studies reported in the literature [12,13]. Therefore, the current findings enhance this understanding by showing that the EO composition of *O. tenuiflorum*, *O. basilicum*, and *O. americanum* can vary depending on the harvesting time. The present study also confirms that seasonal variations have a significant influence on both the quality and quantity of volatile compounds in essential oils.

### 2.3. Enantiomeric Distributions of Essential Oils

Chiral GC-MS is one of the best techniques for confirming the authenticity and uniformity of essential oils. In this study, it was used to identify and analyze the composition of various chiral compounds present in the essential oils of three *Ocimum* species, as shown in Table 4, Table 5 and Table 6.

The chiral terpenoid components determined in the EOs of three *Ocimum* species were 5–6 in *O. tenuiflorum*, 10–12 in *O. americanum,* and 8 in *O. basilicum* from winter to summer for their enantiomeric distributions, respectively. Essential oils extracted from the various plants are sometimes contaminated by the addition of carrier oils or other foreign substances [14,15,16]. However, the enantiomeric ratios of chiral compounds found in the EOs are typically unaffected by the distillation techniques and the geographical origin. Despite having the identical physicochemical property, the (+)- and (–)-enantiomers differ significantly in their biological and organoleptic characteristics. The chiral terpenoids identified in the *O. tenuiflorum* were predominantly levorotatory compounds such as camphene, sabinene, β-pinene, and limonene, whereas the only α-pinene detected in *Ocimum* essential oil was the dextrorotatory enantiomer. This analysis also reveals that compounds such as borneol and β-caryophyllene were identified as enantiomerically pure levorotatory forms in *O. tenuiflorum.* The most dominant enantiomer was (–)-camphene in the EO. The chiral terpenoid components analyzed in *O. americanum* included α-pinene, camphene, β-pinene, limonene, *cis*-sabinene hydrate, linalool, camphor, terpinen-4-ol, borneol, α-terpineol, β-caryophyllene, and germacrene D. Here, β-caryophyllene and germacrene D were found enantiomerically pure in levorotatory to dextrorotatory forms in *O. americanum.* The most predominant chiral compound in this EO was (+)-camphor. In this EO sample, the other compounds, such as α-pinene, camphene, β-pinene, limonene, *cis*-sabinene hydrate, and terpinen-4-ol, were predominantly present in their dextrorotatory forms, while linalool, borneol, and α-terpineol were mainly found in their levorotatory forms. The essential oil of *O. basilicum* was analyzed for enantiomeric distributions of chiral terpenoid compounds, including α-pinene, β-pinene, limonene, linalool, α-terpineol, β-caryophyllene, germacrene D, and β-bisabolene. This study indicates that β-caryophyllene and germacrene D were present as enantiomerically pure forms, ranging from levorotatory to dextrorotatory *in O. basilicum* samples. Among the chiral compounds, (–)-linalool emerged as the most abundant enantiomer in the *O. basilicum* EO. α-Pinene and limonene were found in nearly racemic mixtures, while β-pinene and β-bisabolene were predominantly in dextrorotatory forms. In contrast, linalool, borneol, and α-terpineol were mainly present in their levorotatory forms, with linalool being the most dominant levorotatory enantiomer among them. As far as we know, this is the first report documenting the detailed enantiomeric distribution of chiral compounds in *Ocimum* essential oil, offering valuable insights for the standardization, quality control, and adulteration detection of these commercially important oils.

### 2.4. Hierarchical Cluster Analysis of Ocimum Essential Oils

**Figure 2:**
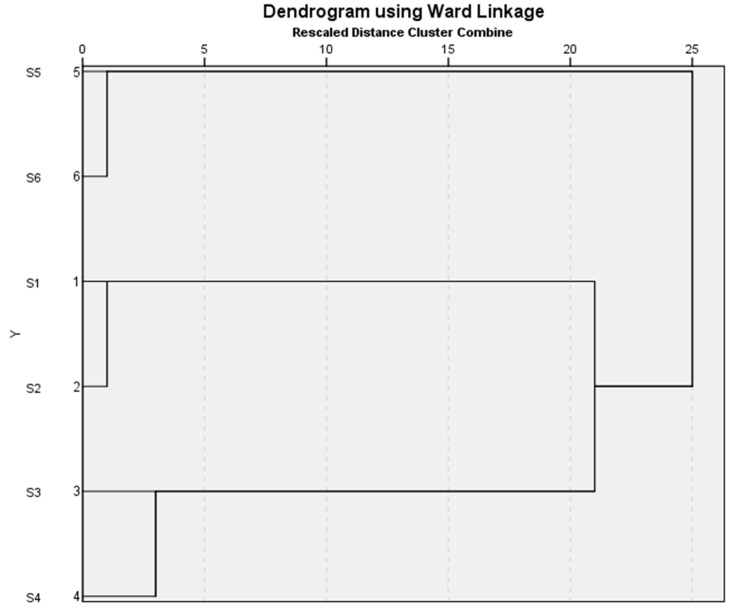
Agglomerative hierarchical cluster (AHC) analysis based on the concentrations of chemical constituents of *Ocimum* essential oil.

Agglomerative hierarchical cluster analysis (HCA) was performed based on the chemical compositions of the *Ocimum* species EOs under this study in order to highlight their major chemotypes (Table S17 in the SM). The dendrogram obtained from this analysis is shown in Figure 2, and HCA clearly indicated that the samples fell into three distinct compositional clusters: methyl chavicol/-linalool (cluster #1), eugenol, trans-β-elemene, and β–caryophyllene (cluster #2), and camphor/-linalool (cluster #3).

### 2.5. Antimicrobial Efficacy

Essential oils of three *Ocimum* species were employed for the evaluation of antimicrobial activity against six different microbial strains and are presented in Table 7 and method as in Figure S7 of SM. In terms of minimum inhibitory concentration (MICs), the antimicrobial potential of *Ocimum* species was shown to be moderately active against all microbial strains when compared to the standard; nevertheless, there was some variance in their efficacies. EOs of three *Ocumum* species demonstrated varying degrees of antimicrobial activity. *O. tenuiflorum* samples collected from Bardiya showed potent antifungal effects against *C. albicans*, with MICs ranging from 162.5 to 325 µg/mL. These samples were rich in key bioactive compounds, notably eugenol (32.14–34.95%), *trans*-β-elemene (29.08–32.85%), and β-caryophyllene (19.22–21.64%). In addition, minor quantities of antimicrobial agents such as camphor, linalool, methyl chavicol, and caryophyllene oxide were also present. Similarly, *O. americanum* EO from the Thankot region showed strong antifungal activity against *C. albicans* with an MIC value of 350 µg/mL. Camphor, the predominant component (51.30–65.88%), is believed to be responsible for this inhibition and is known to induce the expression of the *CDR1* gene in *C. albicans* [17].

The antifungal activity of *O. tenuiflorum* appears to be strongly correlated with its high eugenol content, which is known to inhibit fungus growth through DNA fragmentation [18]. A study on *O. basilicum* from Turkey reported limited antibacterial activity against the strains of *Bacillus*, *Micrococcus*, *Escherichia*, and *Staphylococcus*, with strong inhibition observed only against *Acinetobacter* [19]. In contrast, *O. basilicum* from Kapilvastu, Nepal, showed moderate antimicrobial activity, with MICs between 650 and 2600 µg/mL. The primary constituent in these samples was methyl chavicol, present at a higher level (62.16–64.42%) compared to the samples from Bangladesh (36.7 to 29.9%).

Although the samples from Bangladesh demonstrated strong antimicrobial activity against the food-borne pathogens such as *Bacillus*, *Staphylococcus*, *Listeria*, *Escherichia*, *Shigella*, *Vibrio*, and *Salmonella* with MICs ranging from 62.5 to 500 µg/m, the data suggest that methyl chavicol alone may not be solely responsible for antimicrobial efficacy [12]. Instead, the combined action of multiple constituents appears to contribute to the overall activity of *Ocimum* EOs [20]. Notably, eugenol has been shown to inhibit *Aspergillus niger* [21], and both eugenol and linalool are recognized for their effectiveness against common food-borne pathogens, including *E. coli*, *Salmonella typhimurium*, *Listeria monocytogenes*, and *Vibrio vulnificus*.

### 2.6. Cytotoxic Activity of Essential Oils

**Figure 3:**
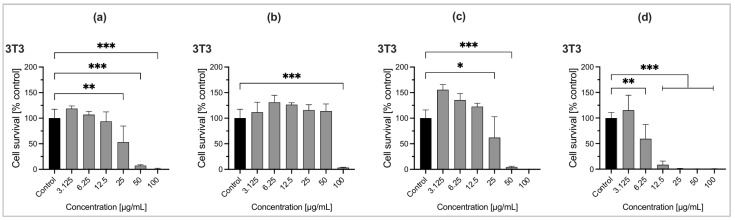
Graph showing the cytotoxicity of essential oils ((**a**). *O. tenuiflorum*, (**b**). *O. basilicum*, (**c**). *O. americanum*) and the reference drug gemcitabine (**d**) against murine fibroblast 3T3 cells in terms of cell survival versus concentrations (µg/mL). Data are expressed as mean ± standard deviation (n = 3). Significance levels: *** *p* < 0.001, ** *p* < 0.01, * *p* < 0.1.

**Figure 4:**
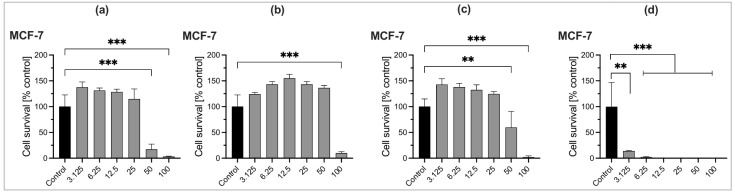
Graph showing the cytotoxicity of essential oils ((**a**). *O. tenuiflorum*, (**b**). *O. basilicum*, (**c**). *O. americanum*) and the reference drug gemcitabine (**d**) against human breast adenocarcinoma MCF-7 cells. Data are expressed as mean ± standard deviation (n = 3). Statistical analysis was performed using ordinary one-way ANOVA followed by multiple comparisons. Significance levels: *** *p* < 0.001, ** *p* < 0.01, * *p* < 0.1.

Cell survival and cytotoxic effects can be reliably determined using the Cell Counting Kit-8 (WST-8/CCK8) method [22]. The cytotoxicity of three *Ocimum* essential oils and the reference drug gemcitabine was investigated against murine fibroblast 3T3 cells and human breast adenocarcinoma MCF-7 cells. This is shown by a plot of cell survival (%) versus logarithm concentrations (µg/mL) as presented in Figure 3 and Figure 4, also Figures S8–S10, in the SM. Gemcitabine was used as a reference drug. Statistical analysis was performed using ordinary one-way ANOVA followed by multiple comparisons. Significance levels are denoted by *** *p* < 0.001, ** *p* < 0.01, and * *p* < 0.1, as shown in Figure 3 and Figure 4. Essential oils at low concentrations (µg/mL) stimulated cell growth, while higher concentrations exhibited cytotoxic effects. Only statistically significant inhibitory effects are shown, while non-significant data are not indicated in the graph.

*Ocimum* species EOs exhibited cytotoxic effects on both cell lines based on changes in concentrations, expressed as IC_50_ values (i.e., 50% cells killed in DMEM). Cytotoxicity of EOs in terms of IC_50_ values is presented in Table 8. At lower concentrations, EO samples may promote proliferation, reflected by the cell index (%). Although some samples exhibit cytotoxicity at high concentrations (e.g., 100 µg/mL), they can stimulate cell growth at lower doses, limiting their potential as anticancer agents. *Ocimum* species EOs showed a weak proliferative effect on MCF-7 cells at low concentrations, as illustrated by the cell index (%) versus the concentration (µM).

Among the three *Ocimum* EOs, *O. tenuiflorum* EO collected from Bardiya during autumn exhibited strong cytotoxic activity against the MCF-7 cancer cell line (IC_50_ = 23.43 µg/mL). The *O. tenuiflorum* has a better selective cytotoxicity, i.e., is more toxic to cancer cells (MCF-7) than normal cells (NIH-3T3) (difference of ~11 µg/mL), indicating a promising anticancer agent. Whereas, *O. americanum* EO taken from Thankot during summer has moderate cytotoxic activity against MCF-7 cell lines (IC_50_ = 57.42 µg/mL) but lacks selectivity, making it less attractive for therapeutic use. Here, three *Ocimum* samples showed relatively weaker anticancer potential compared to the standard, gemcitabine, with IC_50_ values of 0.4977 µg/mL for the MCF-7 cell line and 0.5175 µg/mL for the NIH-3T3 cell line. Moreover, *O. basilicum* EO from Kapilvastu during winter showed weak cytotoxic activity against both MCF-7 and NIH-3T3 cell lines (IC_50_ = 92.88 µg/mL and IC_50_ = 90.56 µg/mL). High IC_50_ values in both cell lines suggest low potency and low selectivity, indicating weak or negligible anticancer activity. The cell viability was markedly reduced at higher concentrations of EOs. As per published guidelines [23], an IC_50_ value between 10 and 100 µg/mL indicates prominent inhibitory capacity against cancer cells, suggesting that these EOs possess notable cytotoxic activity. This cytotoxicity may result from the synergistic interactions between minor constituents and major bioactive compounds [24]. Sesquiterpenes have also been identified as the primary contributors to the cytotoxic effects of these essential oils [25]. Only a limited number of studies have reported on the cytotoxic effects of *Ocimum* species EOs. Thus, among the tested essential oils, *O. tenuiflorum* shows the strongest and most selective anticancer activity, making it the most promising for further investigation. *O. tenuiflorum* EO is less potent than Gemcitabine, but may be potentially safer as a natural source, making it a good agent for alternative therapies with further investigation.

### 2.7. Antioxidant Activity (DPPH and ABTS Assays)

The antioxidant activity of EOs from three *Ocimum* species was determined using DPPH and ABTS radical-scavenging assays. The results were expressed as IC_50_ values, using ascorbic acid, butylated hydroxytoluene (BHT), and quercetin as reference standards (Table 9 and Tables S1–S16 in the SM). These results showed that *O. tenuiflorum* EO collected from Bardiya during autumn exhibited the best antioxidant activity among EOs in DPPH and strong activity in ABTS assays, with IC_50_ values of 69.23 ± 0.10 and 9.05 ± 0.24 μg/mL, respectively. Here, *O. tenuiflorum* EO from Bardiya during winter showed slightly weaker antioxidant activity in DPPH, but the strongest ABTS among Eos, with IC_50_ values of 78.96 ± 0.1 and 5.88 ± 0.80 μg/mL, respectively. *O. basilicum* EO from Kapilvastu during winter showed moderate antioxidant activity in both DPPH and ABTS assays (IC_50s_ = 236.14 ± 0.09 and 44.385 ± 0.81 μg/mL). *O. basilicum* EO (Kapilvastu in summer) had weaker radical-scavenging capacity than the winter sample, showing seasonal variation (IC_50_ values in DPPH and ABTS, 48.21 ± 0.09 and 61.40 ± 0.26 μg/mL), which were lower than reference standards (ascorbic acid, IC_50_ = 6.4 ± 0.3 μg/mL; BHT, IC_50_ = 12.5 ± 0.1 μg/mL; and quercetin, IC_50_ = 7.79 ± 0.65 μg/mL). Furthermore, *O. americanum* essential oil collected from Thankot during summer had the weakest antioxidant activity across both assays (DPPH, IC_50s_ = 452.79 ± 0.90 and ABTS, 145.67 ± 0.20 μg/mL). *O. americanum* EO (Thankot in winter) had slightly better radical-scavenging activity than the summer sample but still weaker activity (IC_50_ values in DPPH and ABTS, 359.17 ± 0.10 and 129.51 ± 1.21 μg/mL). These results suggest low antioxidant potential, indicating *O. americanum* EOs may have fewer phenolic or antioxidant-rich compounds.

Overall, *O. tenuiflorum* essential oil (both winter and autumn) exhibited the strongest radical-scavenging activity, particularly effective in both DPPH and ABTS assays, though not as potent as pure standards. All three *Ocimum* species showed some seasonal variation in antioxidant potential. Winter samples generally outperformed summer ones, possibly due to higher biosynthesis of antioxidant compounds in cooler climates. Although EOs are weaker than synthetic or pure compounds, *O. tenuiflorum* EO still shows promising natural antioxidant potential. The antioxidant qualities of EOs from Lamiaceae plants have been shown in numerous earlier studies; however, no such thorough prior research on the DPPH and ABTS radical-scavenging ability of EOs from *Ocimum* species has been published in the literature.

A previous study reported that essential oils rich in major constituents such as linalool, menthone, and piperitenone oxide demonstrated strong radical-scavenging activity [26]. The superior antioxidant potential and high radical-scavenging efficiency observed in *O. tenuiflorum* and *O. basilicum* essential oils may be attributed to their contents of eugenol, linalool, and methyl chavicol, respectively [27,28,29]. Additionally, another study confirmed that *O. tenuiflorum* essential oil possesses notable radical-scavenging capacity [3].

Previous studies have reported that the antioxidant activity of essential oils may be linked to the presence of mono- and sesquiterpenoids belonging to various major compound classes, including alcohols, ethers, aldehydes, ketones, epoxides, and esters [30]. The moderate radical-scavenging activity observed may result from the enhanced activity of minor constituents or synergistic effects among the components. Moreover, several reports indicate that the EOs as a whole often exhibit stronger radical-scavenging activity than their individual constituents, supporting the notion of synergistic interactions among the various EO components [31]. However, predicting the overall radical-scavenging and antioxidant potential of EOs remains challenging due to their complex composition of multiple active constituents. In this study, the IC_50_ values obtained from the ABTS assay were lower than those from the DPPH assay. This variation can be ascribed to the distinct reaction mechanisms involved in each method. Previous studies have shown that electron-transfer reactions occur more rapidly in the ABTS assay compared to the DPPH assay. In contrast, the DPPH assay primarily reflects the hydrogen-donating capacity of various components within essential oil samples [32]. Therefore, the Bardiya autumn sample has the lowest DPPH IC_50_ among EOs, indicating stronger hydrogen-donating ability and that of Bardiya-winter excels in ABTS scavenging, suggesting excellent electron-donating capacity.

The literature lacks detailed reports on the DPPH radical-scavenging activity of essential oils from many *Ocimum* species. However, the present findings align with certain earlier studies, which demonstrated that essential oils of *O. tenuiflorum* and *O. basilicum* exhibited stronger antioxidant activity than their individual constituents. This suggests a possible synergistic interaction among the components of the essential oils [33,34].

## 3. Materials and Methods

### 3.1. Collection of Plant Materials

**Figure 5:**
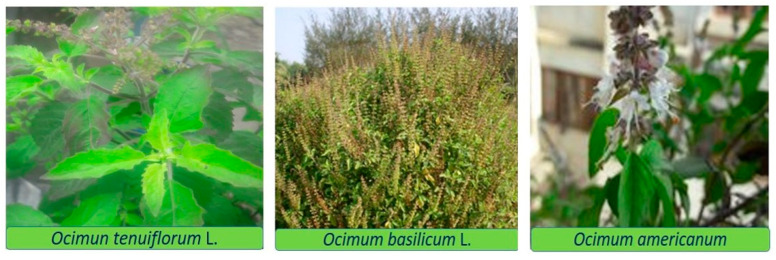
Photographs of three *Ocimum* species taken during sample collection.

**Figure 6:**
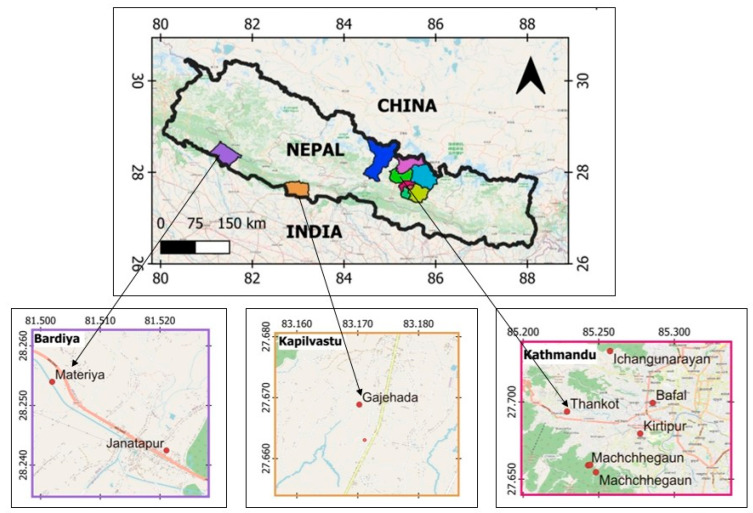
The geographical locations for the collection of three *Ocimum* species from Nepal.

Three species of *Ocimum* (*O. americanum*, *O. tenuiflorum,* and *O. basilicum*) were collected for their aerial parts during the flowering stage in both the winter and summer seasons of 2021 and 2022. The collection sites included Thankot, Kathmandu (elevation: 1383 m; latitude: 27°41′37.7″ N; longitude: 85°13′44.4″ E; 18 December 2021 and 12 June 2022), Bansgadhi, Bardiya (elevation: 159 m; latitude: 28°14′32.9″ N; longitude: 81°31′15.9″ E; 2 December 2021 and 10 October 2022), and Gajehada, Kapilvastu (elevation: 133 m; latitude: 27°40′7.9″ N; longitude: 83°10′12.9″ E; 25 December 2021 and 25 August 2022), as shown in Figure 5 and Figure 6. The collected plant samples were air-dried at room temperature and stored for further analysis. The species identification was carried out by Ms. Rita Chhetri, Senior Research Officer at the National Herbarium and Plant Laboratories (KATH), Lalitpur, Government of Nepal. The herbarium voucher specimens were labeled as BRD-03 for *O. tenuiflorum* L., KTM-04 for *O. americanum* L., and KPV-05 for *O. basilicum* L., respectively.

### 3.2. Essential Oil Extraction

Essential oils were extracted from 100 g of plant material (n = 3) by hydro-distillation using a Clevenger-type apparatus (Jain Scientific Glass Works, JSGW, Haryana, India) [35] for 3 h, following standard methods [36,37], with a plant-to-water ratio of approximately 1:5. The EOs were dried over anhydrous sodium sulfate and stored at 4 °C, and the yield was expressed as a volume-to-weight ratio.

### 3.3. Gas Chromatography–Mass Spectrometry Analysis

The analysis of essential oil samples was performed using the GC-MS technique as previously described [38]. EO components were identified by comparing mass spectral fragmentation patterns (≥80% similarity) and retention indices, determined using n-alkanes (C_8_–C_40_), with literature data [39] and the Aromatic Plant Research Center’s library via LabSolutions GC-MS software v4.45 (Shimadzu Scientific Instruments, Columbia, MD, USA) [40]. Chiral GC-MS analysis followed a previously reported method [41], with enantiomers identified by matching retention times and spectra with Sigma-Aldrich standards (Milwaukee, WI, USA), and enantiomeric ratios calculated from the peak areas.

### 3.4. Hierarchical Cluster Analysis

The essential oil compositions for each sample were treated as operational taxonomic units (OTUs), and the percentages of the most abundant essential oil components were used to establish chemical associations between the essential oil samples using agglomerative hierarchical cluster analysis (HCA) using IBM SPSS STATISTICS VERSION 8.5.5, IBM: Armonk, NY, USA. Dissimilarity was determined using Euclidean distance, and clustering was defined using Ward’s method with automatic entropy truncation.

### 3.5. Antimicrobial Analysis

Antimicrobial potentials of essential oils were evaluated by determining their minimum inhibitory concentration (MICs) by using the micro-broth dilution method against various bacterial strains, like *Bacillus cereus* (ATCC 11778), *Staphylococcus aureus* (ATCC 6538), *Pseudomonas aeruginosa* (ATCC 9027), and *Escherichia coli* (ATCC 8739), and fungal strains *Aspergillus niger* (ATCC 16888) and *Candida albicans* (ATCC 10231), as previously reported [42,43].

### 3.6. Cytotoxicity Analysis

Cytotoxicity of essential oils was evaluated on NIH-3T3 (mouse embryonic fibroblast) and MCF-7 (human breast cancer) cell lines applying the Cell Counting Kit-8 kit (Dojindo, Rockville, MD, USA) in order to assess cell viability. The NIH-3T3 and MCF-7 cell lines (ATCC CRL-1658) were cultured in standard Dulbecco’s Modified Eagle’s medium (DMEM) supplemented with 10% fetal bovine serum (FBS), 0.1% sodium bicarbonate (NaHCO_3_), and 1% antibiotic-antimycotic solution. For cytotoxicity assays, exponentially growing cells were harvested and seeded into 96-well plates at a density of 1 × 10^4^ per well in DMEM. The plates were incubated at 37 °C in a humidified atmosphere containing 5% CO_2_ and 95% air for 24 h. After incubation, cells were washed with PBS and treated with serially diluted test samples in DMEM, including controls and blanks. Following 72 h of incubation, cells were rewashed and incubated with DMEM containing 10% WST-8 solution for 3 h. The absorbance was measured at 450 nm using a Multiscan SkyHigh reader (Thermo Fisher, Waltham, MA, USA), and cell viability was calculated from the mean absorbance of triplicate wells, applying the following Equation (1).

Cell viability (%) = [(Abs(test sample) − Abs(blank))/(Abs(control) − Abs(blank))] × 100%.(1)

Gemcitabine (GEM) was used as the positive control. During the cytotoxic assay, essential oils were tested at concentrations of 100, 50, 25, 12.5, and 6.25 to 3.125 µg/mL. The experiment was run in triplicate, and IC_50_ values were evaluated using non-linear regression of mean ± SD data. Only active samples were selected for analysis prior to CCK-8 addition, with morphological assessment performed at the same point. The IC_50_ value, indicating 50% cell growth inhibition, was determined based on cell viability (live/total cells). EVOS FL (10×) imaging was used to observe cell morphology. The cell index (%) was also assessed, showing that while EOs could kill cells at high concentration (100 µg/mL), they may promote cancer cell growth at lower doses, limiting their potential as anticancer agents.

### 3.7. Antioxidant Potential

#### 3.7.1. DPPH Radical-Scavenging Assay

The DPPH assay was used to assess the free radical-scavenging activity of EOs, with IC_50_ values (μg/mL) determined via non-linear regression [44]. Ascorbic acid, BHT, and quercetin served as reference standards. Experiments were conducted in triplicate.

#### 3.7.2. ABTS Radical-Scavenging Assay

The ABTS assay was used to determine the antioxidant capacities of EO samples as reported previously [45]. Using the given equation [46], the linear % inhibition concentration (IC%) was calculated, and an IC_50_ value was compared with the standard.

### 3.8. Data Analysis

Data were analyzed using Microsoft Excel and OriginPro 2016 64Bit (Origin version 9.3, OriginLab Corporation, ORIGIN: Northampton, MA, USA). Antioxidant and cytotoxicity results were expressed as mean ± standard deviation (SD) from three replicates.

## 4. Conclusions

The present study investigated the essential oils (EOs) from three *Ocimum* species native to Nepal (*O. tenuiflorum*, *O. basilicum*, and *O. americanum*), focusing on their chemical analysis, enantiomeric composition, and biological potentials, including antioxidant, antimicrobial, and cytotoxic effects. The findings clearly showed that harvesting seasons influenced clearly the chemical compositions and biological activities of *Ocimum* EOs. Moreover, the study identified oxygenated monoterpenoids as a major class of volatile organic components. The results revealed that *Ocimum* EOs possess notable antioxidant, antimicrobial, and anticancer properties, particularly *O. tenuiflorum,* highlighting their potential use in pharmaceuticals, food preservation, and cosmetics. However, the potential toxicity of these EOs should be assessed to rectify their safe and effective use. Chiral GC-MS revealed the variation in the enantiomeric distribution of chiral terpenoids, which may serve as important markers for the identification and authentication of *Ocimum* species EOs. Hierarchical cluster analysis (HCA) indicated the presence of three distinct chemotypes within the *Ocimum* species. Overall, *Ocimum* species are valuable sources of bioactive volatile compounds with broad applicability, warranting further investigation to address safety concerns. Additionally, the information on seasonal variation may help to determine the optimal harvesting period for maximum yield and bioactivity. Further research should assess toxicity, clarify mechanisms, link enantiomeric profiles to bioactivity, explore seasonal and chemotype variations, and develop stable, validated formulations for safe pharmaceutical, food, and cosmetic uses.

## Figures and Tables

**Table 1:** The seasonal variation in the chemical composition of *Ocimum tenuiflorum* essential oil.

Lit. RI	Exp. RI	Components	*O. tenuiflorum* (Bardiya-Winter) (%)	*O. tenuiflorum* (Bardiya-Autumn) (%)
Monoterpene hydrocarbons			0.20%	0.31%
932	933	α-Pinene	0.02	0.12
946	950	Camphene	-	0.06
969	972	Sabinene	0.03	0.02
974	978	β-Pinene	0.08	0.05
988	989	Myrcene	0.01	-
1024	1029	Limonene	0.04	0.06
1032	1035	(*Z*)-β-Ocimene	0.01	-
1044	1046	(*E*)-β-Ocimene	0.01	-
Oxygenated monoterpenoids			1.15%	0.99%
1026	1032	1,8-Cineol	0.01	0.06
-	1044	Dihydrotagetone	1.02	-
1095	1099	Linalool	0.02	0.52
1101	1105	*cis*-Thujone	0.03	-
1160	1163	Pinocarvone	0.04	-
1165	1173	Borneol	-	0.41
Sesquiterpene hydrocarbons			57.11%	62.54%
-	1381	*cis*-β-Elemene	1.72	1.92
-	1391	(*E*)-β-Elemene	29.08	32.85
1403	1404	Methyl eugenol	1.21	1.43
1400	1400	β-Longipinene	0.04	-
1408	1405	*cis*-Caryophyllene	-	0.02
1407	1415	α-Barbatene	0.19	0.2
1417	1418	β-Caryophyllene	19.85	21.64
-	1394	epi-Cubebene isomer	-	0.02
1429	1423	*cis*-Thujopsene	-	0.03
1436	1437	Isobazzanene	-	0.08
1440	1447	β-Barbatene	0.22	0.23
1454	1452	(*E*)-β-Farnesene	-	0.06
1452	1454	α-Humulene	1.12	1.18
1476	1483	β-Chamigrene	0.24	0.2
1475	1480	γ-Gurjunene	-	0.03
1489	1489	β-Selinene	1.06	0.81
1496	1499	Valencene	0.04	0.04
1498	1498	α-Selinene	1.01	0.92
1505	1505	α-Cuprenene	-	0.03
1508	1505	Germacrene A	1.19	0.69
1513	1514	γ-Cadinene	0.07	-
1522	1519	δ-Cadinene	-	0.01
1520	1522	7-epi-a-Selinene	-	0.04
-	1523	1,4-Dihydro cuparene	-	0.03
1529	1528	(*E*)-γ-Bisabolene	0.04	0.07
-	1599	Bisabolenol isomer	0.03	-
1532	1534	γ-Cuprenene	-	0.03
Oxygenated sesquiterpenoids			7.28%	1.15%
-	1457	Dehydrosesquicineole	0.02	-
1471	1468	4,5-Di-epi-aristolochene	-	0.02
-	1533	10-epi-Cubenol	0.07	0.02
-	1549	α-Elemol	-	0.04
1582	1583	Caryophyllene oxide	3.37	0.75
1608	1612	Humulene epoxide II	0.35	0.03
1608	1606	β-Atlantol	0.04	-
-	1617	Intermedeol isomer	0.15	0.04
1642	1644	Selina-3,11-dien-6-a-ol	-	0.02
1658	1656	neo-Intermedeol	-	0.19
1639	1634	Caryophylla-4(12),8(13)-dien-5-β-ol	0.14	-
1639	1636	allo-Aromadendrene epoxide	0.05	-
1658	1659	Selin-11-en-4-a-ol	1.45	-
-	1668	Isospathulenol	0.97	0.06
1683	1683	epi-a-Bisabolol	0.04	-
-	1644	Sesquiterpineol	0.63	-
Others			33.53%	35.49%
850	860	(3*Z*)-Hexenol	0.07	0.07
-	896	2,5-Diethyl tetrahydro furan	-	0.01
974	982	1-Octen-3-ol	0.02	0.02
988	996	3-Octanol	-	0.01
1195	1198	Methyl chavicol	0.01	-
-	1294	Dihydroedulan	0.03	-
1306	1357	Eugenol	32.15	34.95
1100	1105	*n*-Nonanal	0.02	-
1436	1437	Isobazzanene	0.08	-
-	1726	Sesquiterpinyl alcohol	1.16	-
-	1846	Phytone	0.03	0.1
-	1879	Phytadiene isomer	-	0.03
2300	2300	Tricosane	-	0.03
2400	2400	Tetracosane	-	0.04
2500	2498	Pentacosane	-	0.05
2600	2600	Hexacosane	-	0.04
2700	2698	Heptacosane	-	0.03
Total			98.28	99.71
	EO yield (%)		0.50 ± 0.05	1.67 ± 0.13

Note: Lit. RI = Literature, Exp. RI = retention index values calculated with respect to a series of *n*-alkanes (C_8_–C_40_) on a ZB-5ms column; components are listed in order of increasing RI values.

**Table 2:** The seasonal variation in the chemical composition of *Ocimum americanum* essential oil.

Lit. RI	Exp. RI	Components	*O. americanum* (Thankot-Winter) (%)	*O. americanum* (Thankot-Summer) (%)
Monoterpene hydrocarbons			11.35%	6.64%
924	925	α-Thujene	0.03	-
932	933	α-Pinene	0.35	0.19
946	950	Camphene	1.44	0.72
969	972	Sabinene	0.09	0.14
974	978	β-Pinene	0.38	0.24
988	989	Myrcene	0.58	0.37
1002	1008	α-Phellandrene	0.12	-
1014	1017	α-Terpinene	0.06	-
1020	1025	p-Cymene	0.18	0.69
1024	1029	Limonene	4.4	3.96
1025	1032	β-Phellandrene	0.04	0.02
1032	1035	(*Z*)-β-Ocimene	0.12	-
1044	1046	(*E*)-β-Ocimene	2.43	0.28
1054	1058	γ-Terpinene	0.25	-
1086	1086	Terpinolene	0.88	0.03
Oxygenated monoterpenoids			68.57%	82.38%
1026	1032	1,8-Cineol	0.31	0.31
-	1044	Dihydrotagetone	0.15	0.09
1065	1071	*cis*-Sabinene hydrate	1.2	1.3
1095	1099	Linalool	9.91	9.72
1122	1127	α-Campholenal	0.18	-
1141	1148	Camphor	51.3	65.88
1159	1155	(*E*)-β-Terpineol	-	0.41
1145	1156	Camphene hydrate	0.3	-
1148	1157	Menthone	0.04	-
1160	1163	Pinocarvone	0.16	-
-	1167	exo-Acetoxy camphene	0.06	-
1165	1173	Borneol	0.46	0.26
1174	1181	Terpinen-4-ol	1.5	1.64
1179	1187	*p*-Cymen-8-ol	-	0.28
1186	1195	α-Terpineol	2.54	1.6
1194	1194	Myrtenol		0.73
-	1202	epi-Borneol	0.18	0.08
-	1179	*p*-1,8-Menthadien-4-ol	0.1	0.13
1239	1242	Carvone	-	0.08
-	1388	Terpenediol	-	0.04
Sesquiterpene hydrocarbons			18.54%	6.75%
1374	1376	α-Copaene	0.56	0.29
1387	1391	β-Bourbonene	0.2	0.13
1387	1388	β-Cubebene	0.16	0.1
-	1391	(*E*)-β-Elemene	0.38	0.19
1417	1418	β-Caryophyllene	6.35	3.97
1430	1431	β-Copaene	0.11	0.03
1452	1454	α-Humulene	0.37	0.27
1484	1484	Germacrene D	7.75	1.38
-	1482	iso-bicyclogeramcrene	1.6	0.3
1500	1496	Bicyclogermacrene	0.59	0.04
1500	1500	α-Muurolene	0.08	-
1513	1514	γ-Cadinene	0.05	-
1522	1519	δ-Cadinene	0.34	0.05
Oxygenated sesquiterpenoids			0.57%	2.58%
-	1549	Isocaryphyllene oxide	-	0.09
1577	1579	Spathulenol	-	0.29
1582	1583	Caryophyllene oxide	0.24	1.91
1608	1612	Humulene epoxide II	-	0.07
1639	1636	allo-Aromadendrene epoxide	-	0.16
1601	1594	(*E*)-β-Elemenone	0.04	-
1638	1643	epi-α-Cadinol	0.11	-
1640	1642	epi-α-Muurolol	0.08	-
1514	1513	γ-Cadinene	0.05	
Others			0.96%	1.49%
850	860	(3*Z*)-Hexenol	0.11	0.27
-	880	2-Butyl furan	-	0.04
-	960	Benzaldehyde	-	0.03
974	982	1-Octen-3-ol	0.1	0.07
988	996	3-Octanol	0.15	0.06
-	1005	Hexenyl acetate	0.1	-
1100	1105	n-Nonanal	0.06	-
1195	1198	Methyl chavicol	0.13	-
-	1183	4-Methyl acetophenone	-	0.06
1179	1187	*p*-Cymen-8-ol	0.06	-
1190	1199	Methyl salicylate	0.07	0.06
1229	1230	(3*Z*)-Hexenyl 2-methyl butanoate	0.04	-
1306	1357	Eugenol	0.21	-
-	1345	2-Methyl-2-(para-tolyl) propionaldehyde	-	0.18
-	1668	Dihydrogermacrene D	-	0.12
1863	1827	*cis*-Thujopsenic acid	-	0.1
1475	1473	trans-Cadina-1(6),4-diene	0.06	-
1565	1571	3-*cis*-Hexenyl benzoate	0.05	-
2300	2301	n-Tricosane	-	0.05
2400	2401	n-Tetracosane	-	0.07
2500	2499	n-Pentacosane	-	0.08
2600	2600	n-Hexacosane	-	0.07
2700	2698	n-Heptacosane	-	0.06
Total			99.89	99.84
	EO yield (%)		0.35 ± 0.02	0.42 ± 0.03

Note: Lit. RI = Literature, Exp. RI = retention index values calculated with respect to a series of n-alkanes (C_8_–C_40_) on a ZB-5ms column; components are listed in order of increasing RI values.

**Table 3:** The seasonal variation in the chemical composition of *Ocimum basilicum* essential oil.

Lit. RT	Exp. RI	Components	*O. basilicum* (Kapilvastu-Summer) (%)	*O. basilicum* (Kapilvastu-Summer) (%)
Monoterpene hydrocarbons			0.71%	0.3%
924	925	α-Thujene	0.02	0.02
932	933	α-Pinene	0.19	0.06
969	972	Sabinene	0.03	0.03
974	978	β-Pinene	0.2	0.04
988	989	Myrcene	0.04	0.04
1024	1029	Limonene	0.02	0.05
1032	1035	(*Z*)-β-Ocimene	-	0.02
1044	1046	(*E*)-β-Ocimene	0.21	0.04
Oxygenated monoterpenoids			28.09%	28.65%
1026	1032	1,8-Cineol	0.05	0.6
-	1044	Dihydrotagetone	0.12	0.03
1067	1071	*cis*-Linalool oxide (furanoid)	0.13	0.14
1084	1087	*trans*-Linalool oxide (furanoid)	0.13	0.18
1083	1092	Fenchone	-	0.02
1095	1099	Linalool	27.05	26.92
-	1131	Limona ketone	0.02	0.03
1186	1195	α-Terpineol	0.01	-
1235	1238	Neral	0.24	0.28
1247	1258	Chavicol	0.02	0.01
1247	1252	*p*-Anis aldehyde	-	0.02
1264	1268	Geranial	0.31	0.11
1282	1291	(*E*)-Anethole	0.12	0.16
1306	1357	Eugenol	0.02	0.02
Sesquiternepe hydrocarbons			5.6%	4.09%
1374	1376	α-Copaene	0.08	0.08
1387	1388	β-Cubebene	0.05	0.09
-	1391	(*E*)-β-Elemene	0.07	0.07
1403	1404	Methyl Eugenol	0.02	0.11
1409	1416	α-Gurjunene	0.02	0.02
1417	1418	β-Caryophyllene	0.79	0.24
1432	1434	*trans*-a-Bergamotene	0.58	0.68
1440	1440	(*Z*)-β-Farnesene	0.08	0.12
1454	1452	(*E*)-β-Farnesene	0.25	0.14
1452	1454	α-Humulene	0.13	0.44
-	1452	epi-Caryophyllene	0.03	0.03
1484	1484	Germacrene D	0.79	0.14
-	1485	*trans*-β-Bergamotene	0.1	-
1500	1496	Bicyclogermacrene	0.09	0.03
1505	1507	β-Bisabolene	0.1	0.02
1522	1519	δ-Cadinene	0.04	0.04
1529	1528	(*E*)-γ-Bisabolene	2.38	1.84
Oxygenated sesquiterpenes			0.44%	1.06%
1515	1515	Sesquicineole	0.01	-
1561	1561	(*E*)-Nerolidol	0.07	0.07
1574	1577	Germacrene D-4-ol	0.04	0.02
1582	1583	Caryophyllene oxide	0.22	0.64
1608	1612	Humulene epoxide II	0.02	-
1649	1655	β-Eudesmol	0.02	0.21
1685	1685	α-Bisabolol	0.06	0.12
Others			64.9%	62.49%
846	856	(*2E*)-Hexenal	0.01	-
-	960	Benzaldehyde	0.01	0.05
981	986	6-Methyl-5-hepten-2-one	0.04	0.01
998	1005	n-Octanal	0.11	0.16
-	1005	Hexenyl acetate	0.03	0.03
-	1101	6-Methyl-3,5-heptadien-2-one	0.01	0.01
1110	1107	1-Octen-3-yl acetate	0.01	-
1195	1198	Methyl chavicol	64.42	62.16
1211	1211	Octyl acetate	0.06	0.01
1384	1375	(3*Z*)-Hexenyl-(3*Z*)-hexenoate	0.15	0.04
-	1559	(*E*)-*p*-Methoxycinnamaldehyde	0.05	0.02
Total			99.74	96.59
	EO yield (%)		0.66 ± 0.09	0.88 ± 0.09

Note: Lit. RI = Literature, Exp. RI = retention index values calculated with respect to a series of n-alkanes (C_8_–C_40_) on a ZB-5ms column; components are listed in order of increasing RI values.

**Table 4:** Enantiomeric distributions of chiral compounds in EOs of *O. tenuiflorum*.

Compounds	RT	RT	*O. tenuiflorum* (Bardiya-Winter) (%)		*O. tenuiflorum* (Bardiya-Autumn) (%)	
	(+)	(−)	(+)	(−)	(+)	(−)
α-Pinene	16.40	15.92	64.32	35.68	53.62	46.38
Sabinene	19.74	20.60	20.8	79.2	-	-
Camphene	18.30	17.73	-	-	2.72	97.28
β-Pinene	20.27	20.62	33.56	66.44	19.66	80.34
Limonene	25.99	25.06	17.14	82.86	38.12	61.88
Borneol	59.11	58.59	-	-	0	100
β-Caryophyllene	NA	69.33	0	100	0	100

Note: RT = Retention time (min), NA = Reference enantiomer not available.

**Table 5:** Enantiomeric distributions of chiral compounds in EOs of *O. americanum*.

Compounds	RT	RT	*O. americanum* (Thankot-Winter) (%)		*O. americanum* (Thankot-Summer) (%)	
	(+)	(−)	(+)	(−)	(+)	(−)
α-Pinene	16.40	15.92	71.59	28.41	67.83	32.17
Camphene	18.30	17.73	91.39	8.61	93.19	8.81
β-Pinene	20.27	20.62	64.97	35.03	55.41	44.59
Limonene	25.99	25.06	82.63	17.37	75.56	23.44
*cis*-Sabinene hydrate	40.70	41.25	-	-	91.23	8.77
Linalool	44.69	45.30	10.27	89.73	1.36	98.64
Camphor	50.12	49.31	99.33	0.67	100	0
Terpinen-4-ol	54.64	54.93	79.85	20.15	76.04	23.96
Borneol	59.11	58.59	-	-	7.4	92.6
α-Terpineol	60.58	59.73	34.59	65.41	44.27	55.73
β-Caryophyllene	NA	69.33	0	100	0	100
Germacrene D	73.48	73.73	0	100	0	100

Note: RT = Retention time (min), NA = Reference enantiomer not available.

**Table 6:** Enantiomeric distributions of chiral compounds in EOs of *O. basilicum*.

Compounds	RT	RT	*O. basilicum* (Kapilvastu-Summer) (%)		*O. basilicum* (Kapilvastu-Winter) (%)	
	(+)	(−)	(+)	(−)	(+)	(−)
α-Pinene	16.40	15.92	55.74	44.26	56.08	43.92
β-Pinene	20.27	20.62	78.89	21.11	57.35	42.65
Limonene	25.99	25.06	49.05	50.95	49.45	50.55
Linalool	44.69	45.30	0.14	99.86	2.12	97.88
α-Terpineol	60.58	59.73	21.75	78.25	25.32	74.68
β-Caryophyllene	69.33	NA	0	100	0	100
Germacrene D	73.48	73.73	0	100	0	100
β-Bisabolene	75.55	75.73	91.71	8.29	92.20	7.80

Note: RT = Retention time (min), NA = Reference enantiomer not available.

**Table 7:** Minimum inhibitory concentrations (MICs) of three *Ocimum* species essential oils against tested bacterial and fungal strains.

Name of Micro-Organism	*O. tenuiflorum*		*O* *. americanum*		*O. basilicum*	
	MICs (µg/mL)					
	(Bardiya- Winter)	(Bardiya-Autumn)	(Thankot-Summer)	(Thankot-Winter)	(Kapilvastu-Summer)	(Kapilvastu-Winter)
*Bacillus cereus* (ATCC 11778)	650	1300	650	1300	1579	1314
*Staphylococcus aureus* (ATCC 6538)	1300	650	650	1300	1257	1579
*Pseudomonas aeruginosa* (ATCC 9027)	1300	650	650	650	1300	650
*Escherichia coli* (ATCC 8739)	650	1300	1300	2600	2628	1530
*Candida albicans* (ATCC 10231)	162.5	325	350	650	1314	1579
*Aspergillusniger* (ATCC 16888)	650	1300	650	1300	2600	2557

Note: Gentamicin (MICs < 19.5) and amphotericin B (MICs < 19.5) were used as positive controls for bacteria and fungi, respectively.

**Table 8:** Cytotoxic activity of three *Ocimum* species and standard against MCF-7 and NIH-3T3 cell lines.

EO Samples and Standard	IC_50_ (µg/mL)	IC_50_ (µg/mL)
	NIH-3T3 Cell Line	MCF-7 Cell Line
O. tenuiflorum (Bardiya-Autumn)	34.62	23.43
O. basilicum (Kapilvastu-Winter)	90.56	92.88
O. americanum (Thankot-Summer)	49.45	57.42
Gemcitabine	0.5175	0.4977

Note: IC_50_ is median inhibitory concentration.

**Table 9:** Antioxidant activity of three *Ocimum* species, ascorbic acid, BHT, and quercetin (standards).

EO Samples and Standard	DPPH Radical-Scavenging IC_50_ Value (µg/mL)	ABTS Radical-Scavenging IC_50_ Value (µg/mL)
*O. tenuiflorum* (Bardiya-Winter)	78.96 ± 0.1	5.88 ± 0.80
*O. tenuiflorum* (Bardiya-Autumn)	69.23 ± 0.10	9.05 ± 0.24
*O. americanum* (Thankot-Winter)	359.17 ± 0.10	129.51 ± 1.21
*O. americanum* (Thankot-Summer)	452.79 ± 0.90	145.67 ± 0.20
*O. basilicum* (Kapilvastu-Summer)	448.21 ± 0.09	61.40 ± 0.26
*O. basilicum* (Kapilvastu-Winter)	236.14 ± 0.09	44.38 ± 0.81
Ascorbic acid	6.37 ± 0.34	1.98 ± 1.20
BHT	12.46 ± 0.09	-
Quercetin	-	7.79 ± 0.65

Note: Values are mean ± standard deviations from three experiments (n = 3).

## Data Availability

All data are available in the manuscript.

## Supplementary Materials

The following supporting information can be downloaded at: https://www.mdpi.com/article/10.3390/molecules30173581/s1, Figure S1: Typical GC-MS chromatogram of *O. tenuiflorum* L. essential oil (Bardiya-winter); Figure S2: Typical GC-MS chromatogram of *O. basilicum* L. essential oil (Kapilvastu-winter); Figure S3: Typical GC-MS chromatogram of *O. americanum* L. essential oil (Thankot-winter); Figure S4: Typical GC-MS chromatogram of *O. americanum* L. essential oil (Thankot-summer); Figure S5: Typical GC-MS chromatogram of *O. tenuiflorum* L. essential oil (Bardiya-autumn); Figure S6: Typical GC-MS chromatogram of *O. basilicum* L. essential oil (Kapilvastu-summer); Figure S7: A flow chart showing the *In-vitro* antimicrobial activity using micro-broth dilution assay for the evaluation of minimum inhibitory concentration (MICs): EO samples (Following standard protocol; Figure S8: Graph showing percent cell survival versus logarithm of the concentration (µg/mL) for cytotoxic activity of (a) *O. tenuiflorum*, (b) *O. basilicum,* (c) *O. americanum,* and (d) standard gemcitabine (Gem-1 and Gem-2) against NIH-3T3 cell line; Figure S9: Graph showing percent cell survival versus logarithm of the concentration (µg/mL) for cytotoxic activity of (a) *O. tenuiflorum*, (b) *O. basilicum,* (c) *O. americanum,* and (d) standard gemcitabine against MCF-7 cell line; Figure S10: Graph showing cell index (%) versus the concentration (µM) for cell proliferation of (a) *O. tenuiflorum*, (b) *O. basilicum,* (c) *O. americanum,* and (d) standard gemcitabine against human breast cancer cell line (MCF-7); Table S1: Average percentage DPPH free radical-scavenging activity of *O. tenuiflorum* EO sample (Bardiya-winter); Table S2: Average percentage DPPH free radical-scavenging activity of *O. tenuiflorum* EO sample (Bardiya-autumn); Table S3: Average percentage DPPH free radical-scavenging activity of *O. basilicum* EO sample (Kapilvastu-winter);Table S4: Average percentage DPPH free radical-scavenging activity of *O. basilicum* EO sample (Kapilvastu-summer); Table S5: Average percentage DPPH free radical-scavenging activity of *O. americanum* EO sample (Thankot-winter); Table S6: Average percentage DPPH free radical-scavenging activity of *O. americanum* EO sample (Thankot-summer); Table S7: Average percentage DPPH free radical-scavenging activity of standard reference (Ascorbic acid); Table S8: Average percentage DPPH free radical-scavenging activity of standard reference (BHT); Table S9: Average percentage ABTS free radical-scavenging activity of *O. tenuiflorum* EO sample (Bardiya-winter); Table S10: Average percentage ABTS free radical-scavenging activity of *O. tenuiflorum* EO sample (Bardiya-autumn);Table S11: Average percentage ABTS free radical-scavenging activity of *O. basilicum* EO sample (Kapilvastu-summer);Table S12: Average percentage ABTS free radical-scavenging activity of *O. basilicum* EO sample (Kapilvastu-winter); Table S13: Average percentage ABTS free radical-scavenging activity of *O. americanum* EO sample (Thankot-winter); Table S14: Average percentage ABTS free radical-scavenging activity of *O. americanum* EO sample (Thankot-summer); Table S15: Average percentage ABTS free radical-scavenging activity of standard reference (Quercetin); Table S16: Average percentage ABTS free radical-scavenging activity of standard reference (Ascorbic acid); Table S17: Agglomerative hierarchical cluster (AHC) analysis based on the concentrations of chemical constituents of three *Ocimum* essential oil.

## Abbreviations

The following abbreviations are used in this manuscript:

GC-MS	Gas Chromatography–Mass Spectrometry
EOs	Essential Oils
DPPH	2,2-diphenyl-1-picrylhydrazyl
ABTS	2,2′-azinobis-3-ethylbenzothiazoline-6-sulfonic acid
IC_50_	Inhibitory Concentration at 50%
RI	Retention Index
RSA	Radical Scavenging Activity
ATCC	American Type Culture Collection
DMSO	Dimethyl Sulphoxide
SM	Supplementary Materials
BHT	Butylated Hydroxytoluene
HCA	Hierarchial Cluster Analysis
MIC	Minimum Inhibitory Concentration
DMEM	Dulbecco’s Modified Eagle Medium
CAMHB	Cation-adjusted Muller Hinton Broth
PBS	Phosphate-Buffered Saline
