# Supplementary material for: Seasonal Variation in Essential Oil Composition and Bioactivity of Three Ocimum Species from Nepal
Source: Molecules. 2025 Sep 1;30(17):3581. doi: 10.3390/molecules30173581 (PMC12430450; doi:10.3390/molecules30173581)
Supplement: Supplementary file 1 [file molecules-30-03581-s001.zip › molecules-3774511-supplementary.pdf]

Supplementary Materials

I.D. No.: molecules-3774511

Article Title:

Seasonal Variation in Essential Oil Composition and Bioactivity of Three *Ocimum* Species from Nepal

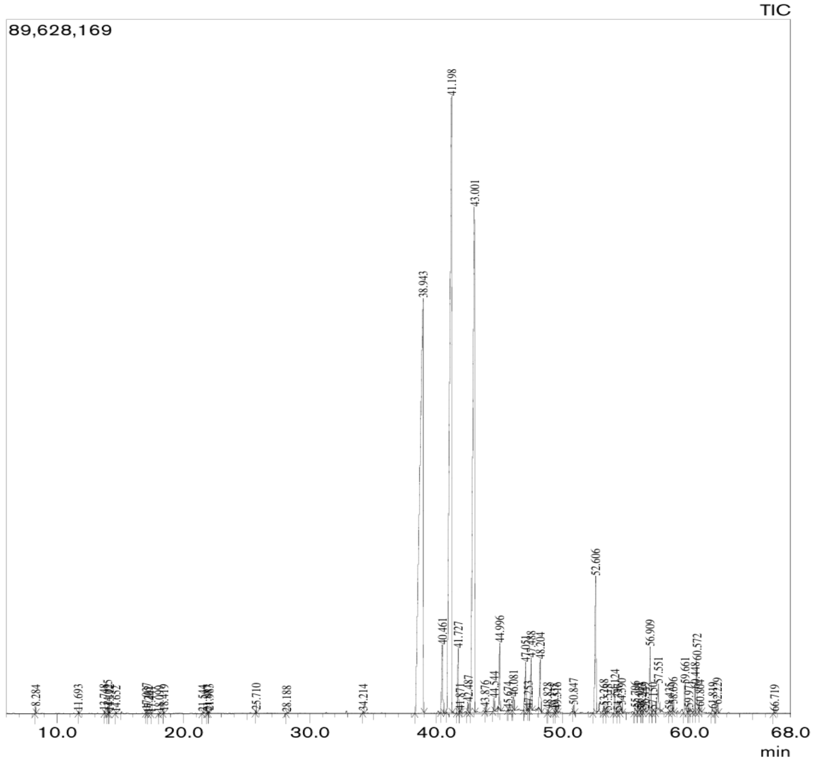

Figure S1. Typical GC-MS chromatogram of *O. tenuiflorum* L. essential oil (Bardiya-winter)

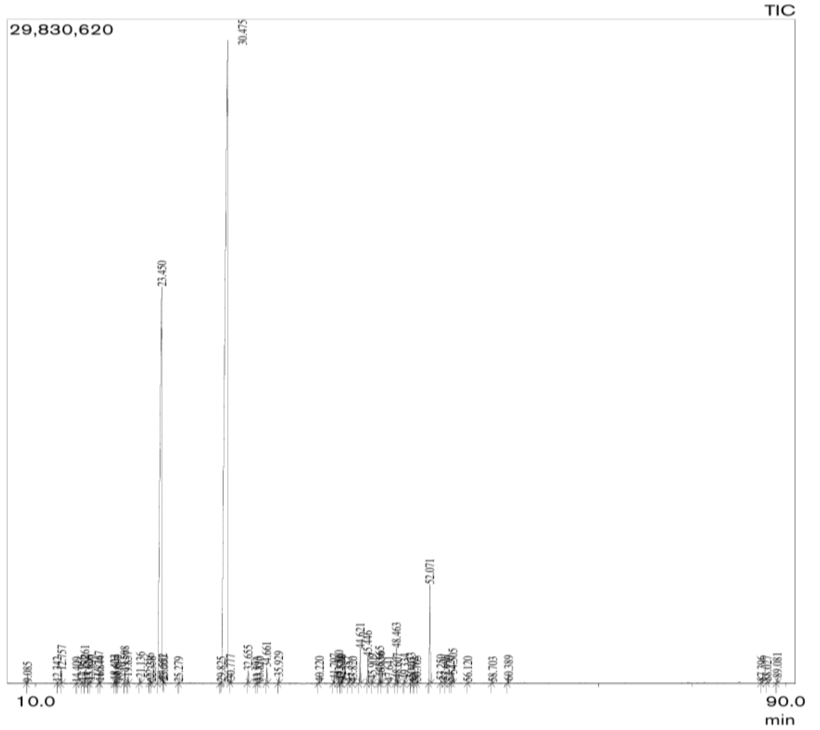

Figure S2. Typical GC-MS chromatogram of *O. basilicum* L. essential oil (Kapilvastu-winter)

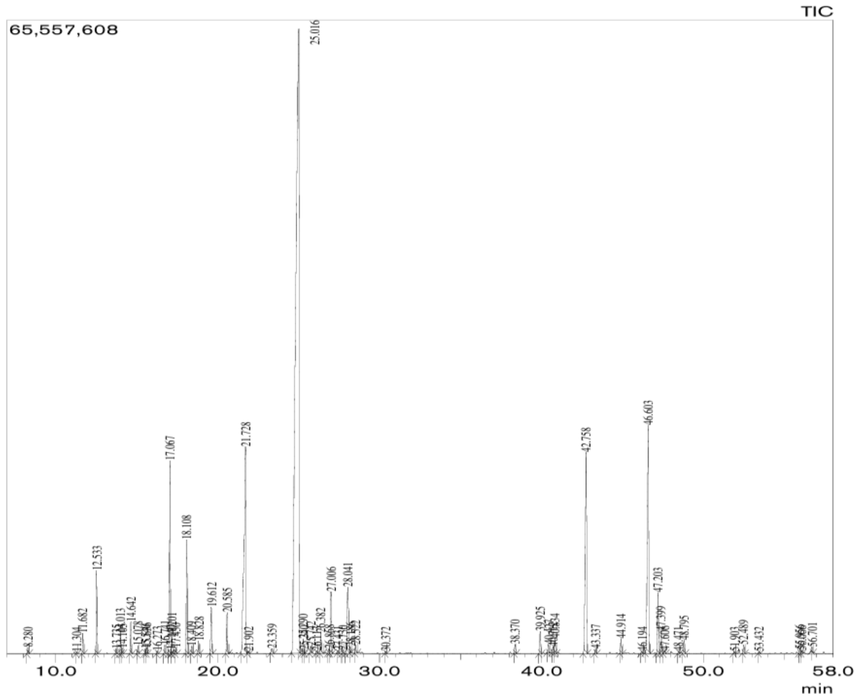

Figure S3. Typical GC-MS chromatogram of *O. americanum* L. essential oil (Thankot-winter)

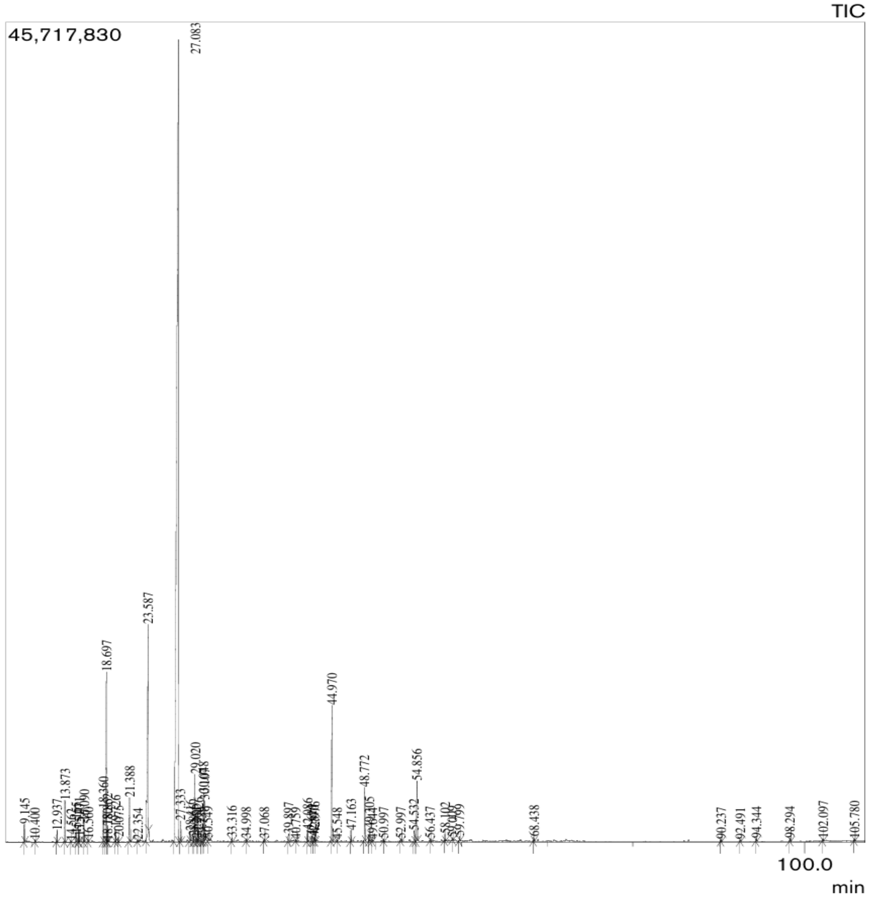

Figure S4. Typical GC-MS chromatogram of *O. americanum* L. essential oil (Thankot-summer)

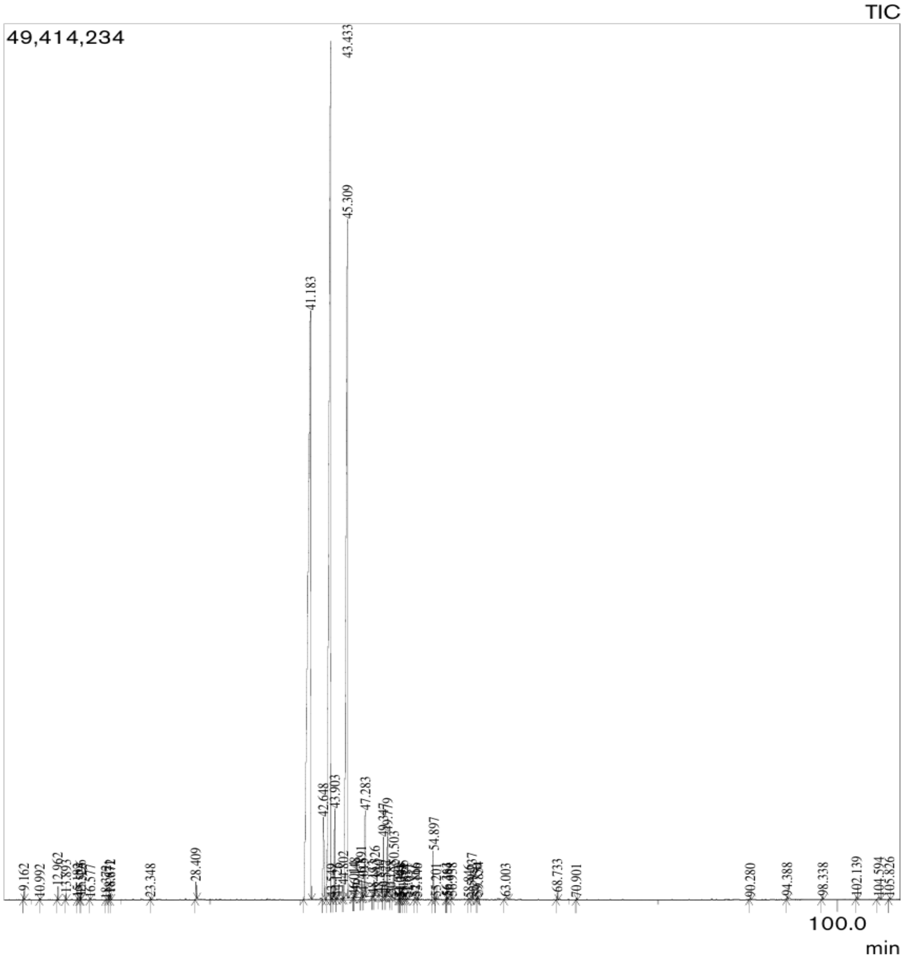

Figure S5. Typical GC-MS chromatogram of *O. tenuiflorum* L. essential oil (Bardiya-autumn)

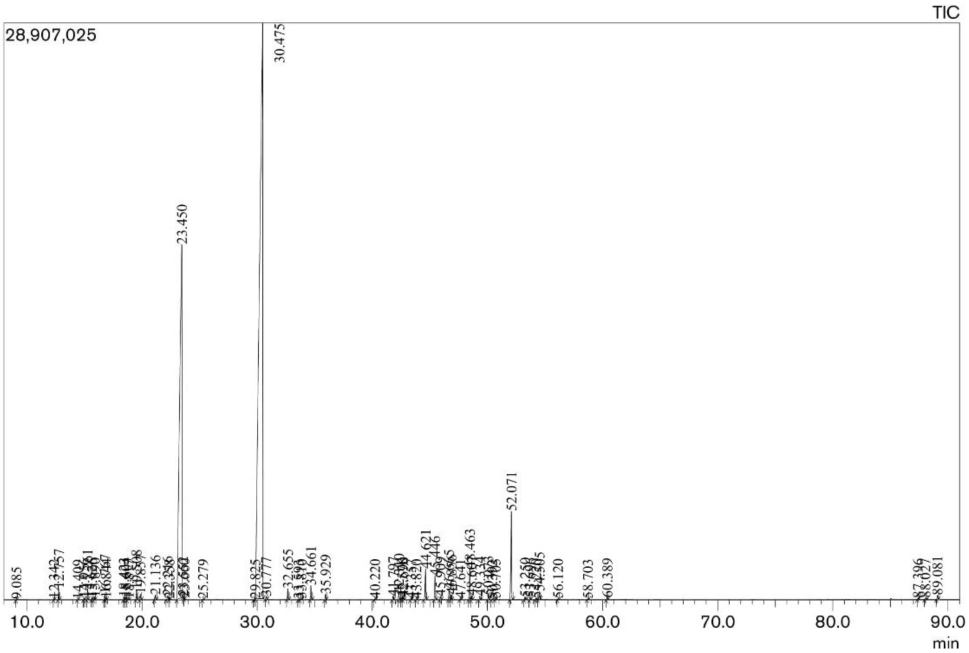

Figure S6. Typical GC-MS chromatogram of *O. basilicum* L. essential oil (Kapilvastu-summer)

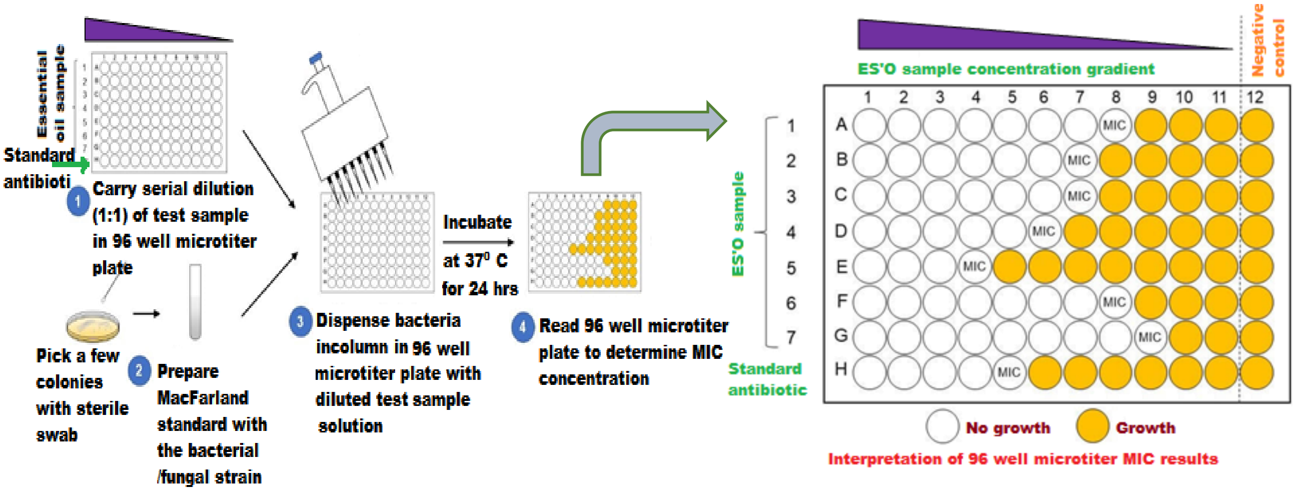

Figure S7. A flow chart showing the *In-vitro* antimicrobial activity using micro-broth dilution assay for the evaluation of minimum inhibitory concentration (MICs): EO samples (Following standard protocol, Ref. 42,43)

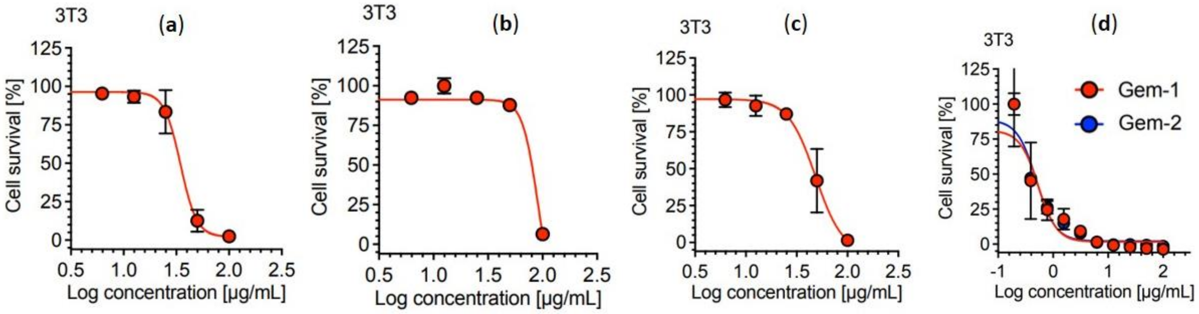

Figure S8. Graph showing percent cell survival versus logarithm of the concentration (µg/mL) for cytotoxic activity of (a) *O. tenuiflorum*, (b) *O. basilicum*, (c) *O. americanum*, and (d) standard gemcitabine (Gem-1 and Gem-2) against NIH-3T3 cell line.

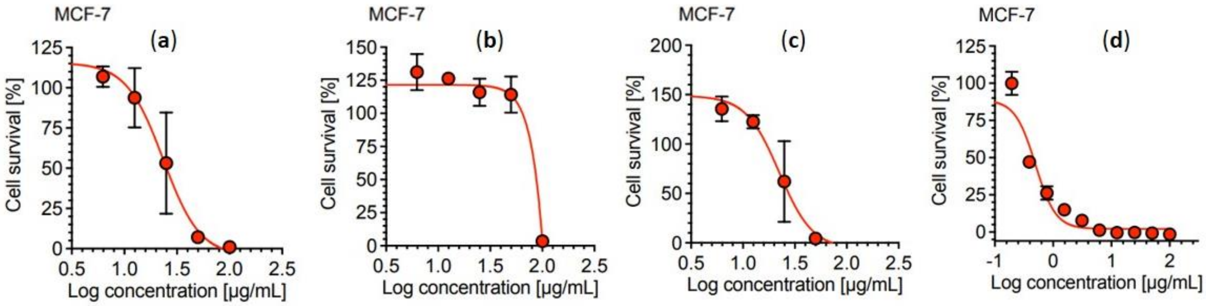

Figure S9. Graph showing percent cell survival versus logarithm of the concentration (µg/mL) for cytotoxic activity of (a) *O. tenuiflorum*, (b) *O. basilicum*, (c) *O. americanum*, and (d) standard gemcitabine against MCF-7 cell line.

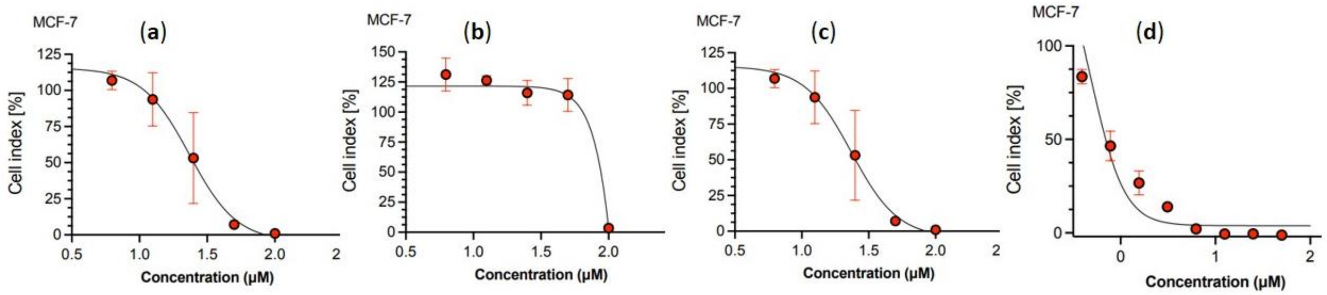

Figure S10. Graph showing cell index (%) versus the concentration (µM) for cell proliferation of (a) *O. tenuiflorum*, (b) *O. basilicum*, (c) *O. americanum*, and (d) standard gemcitabine against human breast cancer cell line (MCF-7).

Table S1. Average percentage DPPH free radical-scavenging activity of *O. tenuiflorum* EO sample (Bardiya-winter)

S. N.	Conc. (µg/mL)	R1	R2	R3	Average of R1, R2, R3	Average ± S.D.	% Scavenging activity
1.	1000	0.06	0.06	0.064	0.061333	0.06±0.03	82.47619
2.	500	0.164	0.162	0.166	0.164000	0.06±0.14	81.33333
3.	250	0.064	0.065	0.067	0.065333	0.06±0.03	73.06792
4.	125	0.124	0.126	0.125	0.125000	0.13±0.06	64.28571
5.	62.5	0.198	0.200	0.198	0.198667	0.20±0.04	43.23810
y=0.1936x+34.714; IC_{50} = 78.96±0.12 (µg/mL)							

Table S2. Average percentage DPPH free radical-scavenging activity of *O. tenuiflorum* EO sample (Bardiya-autumn)

S. N.	Conc. (µg/mL)	R1	R2	R3	Average of R1, R2, R3	Average ± S.D.	% Scavenging activity
1.	1000	0.050	0.050	0.040	0.046667	0.05±0.01	87.62157
2.	500	0.052	0.054	0.056	0.054000	0.05±0.12	85.67639
3.	250	0.109	0.109	0.111	0.109667	0.11±0.04	70.91070
4.	125	0.159	0.158	0.156	0.157667	0.16±0.09	58.17860
5.	62.5	0.211	0.213	0.211	0.211667	0.21±0.12	43.8550
y=0.0887x+43.859; IC_{50} = 69.23±0.10 (µg/mL)							

Table S3. Average percentage DPPH free radical-scavenging activity of *O. basilicum* EO sample (Kapilvastu-winter)

S. N.	Conc. (µg/mL)	R1	R2	R3	Average of R1, R2, R3	Average ± S.D.	% Scavenging activity
1.	1000	0.101	0.100	0.098	0.099667	0.10±0.03	71.52381
2.	500	0.122	0.125	0.126	0.124333	0.12±0.08	64.47619
3.	250	0.166	0.165	0.164	0.165	0.17±0.02	52.85714
4.	125	0.185	0.188	0.186	0.186333	0.19±0.02	46.7619
5.	62.5	0.222	0.231	0.224	0.225667	0.23±0.11	35.52381
y=0.0599x+35.855; IC_{50} = 236.14±0.09 (µg/mL)							

Table S4. Average percentage DPPH free radical-scavenging activity of *O. basilicum* EO sample (Kapilvastu-summer)

S. N.	Conc. (μg/mL)	R1	R2	R3	Average of R1, R2, R3	Average ± S.D.	% Scavenging activity
1.	1000	0.121	0.126	0.128	0.12500	0.13±0.06	64.28571
2.	500	0.164	0.165	0.168	0.165667	0.17±0.02	52.66667
3.	250	0.188	0.189	0.187	0.188000	0.19±0.08	46.28571
4.	125	0.203	0.200	0.201	0.201333	0.20±0.03	42.47619
5.	62.5	0.222	0.224	0.228	0.224667	0.22±0.02	35.80952
y=0.0278x+37.54; IC_{50} = 448.21±0.09 (μg/mL)							

Table S5. Average percentage DPPH free radical-scavenging activity of *O. americanum* EO sample (Thankot-winter)

S. N.	Conc. (μg/mL)	R1	R2	R3	Average of R1, R2, R3	Average ± S.D.	% Scavenging activity
1.	1000	0.123	0.127	0.121	0.123667	0.12±0.09	67.19717
2.	500	0.155	0.159	0.158	0.157333	0.16±0.02	58.26702
3.	250	0.209	0.2	0.201	0.203333	0.20±0.07	46.06543
4.	125	0.245	0.249	0.248	0.247333	0.25±0.02	34.39434
5.	62.5	0.281	0.285	0.27	0.278667	0.29±0.18	26.08311
y=0.0707x+24.607; IC_{50} = 359.16±0.11 (μg/mL)							

Table S6. Average percentage DPPH free radical-scavenging activity of *O. americanum* EO sample (Thankot-summer)

S. N.	Conc. (μg/mL)	Average	Average ± S.D.	% Scavenging activity
1.	1000	0.100	0.100±0.09	84.61538462
2.	750	0.140	0.140±0.08	72.30769231
3.	500	0.192	0.192±0.01	56.30769231
4.	250	0.256	0.256±0.14	36.61538462
5.	125	0.289	0.289±0.1	26.46153846
6.	62.5	0.304	0.304±0.11	21.84615385
IC_{50} = 452.79±0.90 (μg/mL)				

Table S7. Average percentage DPPH free radical-scavenging activity of standard reference (Ascorbic acid)

S. N.	Conc. (μg/mL)	R1	R2	R3	Average of R1, R2, R3	Average ± S.D.	% Scavenging activity
1.	20	0.060	0.064	0.062	0.062	0.062±0.01	83.68421
2.	10	0.064	0.068	0.069	0.067	0.067±0.02	82.36842
3.	5	0.217	0.214	0.217	0.216	0.216±0.00	43.15789
4.	2.5	0.318	0.324	0.322	0.321	0.321±0.01	15.4386
5.	1.25	0.374	0.375	0.378	0.376	0.376±0.00	1.140351
Y=9.2021x-7.6087; IC_{50} = 6.37±0.34 (μg/mL)							

Table S8. Average percentage DPPH free radical-scavenging activity of standard reference (BHT)

S. N.	Conc. (μg/mL)	Average	Average ± S.D.	% Scavenging activity
1.	20	0.14	0.14±0.12	72.30769
2.	15	0.162	0.162±0.07	65.53846
3.	10	0.234	0.234±0.02	43.38462
4.	7.5	0.269	0.269±0.09	32.61538
5.	5	0.308	0.308±0.04	20.61538
6.	3.75	0.324	0.324±0.1	15.69231
IC_{50} = 12.46±0.09 (μg/mL)				

Table S9. Average percentage ABTS free radical-scavenging activity of *O. tenuiflorum* EO sample (Bardiya-winter)

S. N.	Conc. (μg/mL)	R1	R2	R3	Average of R1, R2, R3	Average ± S.D.	% Scavenging activity
1.	1000	0.104	0.105	0.103	0.104	0.104± 0.001	84.906
2.	500	0.056	0.057	0.061	0.058	0.058± 0.003	91.582
3.	250	0.049	0.051	0.056	0.052	0.052± 0.004	92.453
4.	125	0.049	0.054	0.051	0.051	0.051± 0.003	92.550
5.	62.5	0.05	0.049	0.052	0.050	0.050± 0.002	92.695
6.	31.25	0.08	0.082	0.084	0.082	0.082± 0.002	88.099
7.	15.625	0.189	0.188	0.192	0.190	0.190± 0.002	72.472
8.	7.8125	0.301	0.316	0.311	0.309	0.309± 0.008	55.104
9.	3.90625	0.402	0.405	0.406	0.404	0.404± 0.002	41.316
y=1.6249x+40.445; IC_{50} = 5.88±0.80 (μg/mL)							

Table S10. Average percentage ABTS free radical-scavenging activity of *O. tenuiflorum* EO sample (Bardiya-autumn)

S. N.	Conc. (μg/mL)	R1	R2	R3	Average of R1, R2, R3	Average ± S.D.	% Scavenging activity
1.	1000	0.096	0.102	0.096	0.098	0.098± 0.003	85.776
2.	500	0.071	0.068	0.066	0.068	0.068± 0.003	90.082
3.	250	0.055	0.05	0.044	0.050	0.050± 0.006	92.791
4.	125	0.047	0.045	0.046	0.046	0.046± 0.001	93.324
5.	62.5	0.046	0.047	0.053	0.049	0.049± 0.004	92.937
6.	31.25	0.09	0.092	0.094	0.092	0.092± 0.002	86.647
7.	15.625	0.194	0.196	0.196	0.195	0.195± 0.001	71.650
8.	7.8125	0.348	0.348	0.346	0.347	0.347± 0.001	49.589
9.	3.90625	0.489	0.488	0.482	0.486	0.486± 0.004	29.415
y=3.4926x+18.384; IC_{50} = 9.05±0.24 (μg/mL)							

Table S11. Average percentage ABTS free radical-scavenging activity of *O. basilicum* EO sample (Kapilvastu-summer)

S. N.	Conc. (µg/mL)	R1	R2	R3	Average of R1, R2, R3	Average ± S.D.	% Scavenging activity
1.	1000	0.09	0.087	0.087	0.088	0.088±0.002	87.302
2.	500	0.094	0.096	0.092	0.094	0.094±0.002	86.436
3.	250	0.107	0.119	0.147	0.124	0.124±0.021	82.059
4.	125	0.242	0.233	0.243	0.239	0.239±0.006	65.464
5.	62.5	0.318	0.328	0.342	0.329	0.329±0.012	52.477
6.	31.25	0.386	0.392	0.392	0.390	0.390±0.003	43.723
7.	15.625	0.452	0.442	0.45	0.448	0.448±0.005	35.354
y=0.2618x+33.915; IC_{50} = 61.40±0.26 (µg/mL)							

Table S12. Average percentage ABTS free radical-scavenging activity of *O. basilicum* EO sample (Kapilvastu-winter)

S. N.	Conc. (µg/mL)	R1	R2	R3	Average of R1, R2, R3	Average ± S.D.	% Scavenging activity
1.	1000	0.068	0.071	0.075	0.071	0.071±0.004	89.647
2.	500	0.117	0.123	0.119	0.120	0.120±0.003	82.632
3.	250	0.183	0.191	0.189	0.188	0.188±0.004	72.762
4.	125	0.223	0.234	0.232	0.230	0.230±0.006	66.667
5.	62.5	0.301	0.284	0.289	0.291	0.291±0.009	57.716
6.	31.25	0.384	0.388	0.384	0.385	0.385±0.002	44.074
7.	15.625	0.421	0.425	0.43	0.425	0.425±0.005	38.268
y=0.418x+31.447; IC_{50} = 44.38±0.81 (µg/mL)							

Table S13. Average percentage ABTS free radical-scavenging activity of *O. americanum* EO sample (Thankot-winter)

S. N.	Conc. (µg/mL)	R1	R2	R3	Average of R1, R2, R3	Average ± S.D.	% Scavenging activity
1.	1000	0.072	0.065	0.065	0.067	0.067±0.004	90.284
2.	500	0.123	0.124	0.093	0.113	0.113±0.018	83.646
3.	250	0.214	0.214	0.189	0.206	0.206±0.014	70.322
4.	125	0.344	0.352	0.371	0.356	0.356±0.014	48.677
5.	62.5	0.401	0.412	0.411	0.408	0.408±0.006	41.126
y=0.1582x+30.303; IC_{50} = 129.51±1.21 (µg/mL)							

Table S14. Average percentage ABTS free radical-scavenging activity of *O. americanum* EO sample (Thankot-summer)

S. N.	Conc. (µg/mL)	R1	R2	R3	Average of R1, R2, R3	Average ± S.D.	% Scavenging activity
1.	1000	0.06	0.064	0.062	0.062	0.062±0.002	91.053
2.	500	0.156	0.203	0.193	0.184	0.184±0.025	73.295
3.	250	0.254	0.258	0.255	0.256	0.256±0.002	62.893
4.	125	0.351	0.358	0.355	0.355	0.355±0.004	48.524
5.	62.5	0.412	0.444	0.412	0.423	0.423±0.018	38.655
y=0.1272x+31.471; IC_{50} = 145.66±0.20 (µg/mL)							

Table S15. Average percentage ABTS free radical-scavenging activity of standard reference (Quercetin)

S. N.	Conc. (μg/mL)	R1	R2	R3	Average of R1, R2, R3	Average ± S.D.	% Scavenging activity
1.	250	0.061	0.061	0.068	0.063	0.063±0.004	90.861
2.	125	0.077	0.074	0.089	0.080	0.080±0.008	88.456
3.	62.5	0.074	0.102	0.073	0.083	0.083±0.002	88.023
4.	31.25	0.111	0.108	0.107	0.109	0.109±0.016	84.319
5.	15.625	0.231	0.217	0.208	0.219	0.219±0.012	68.446
6.	7.8125	0.329	0.322	0.328	0.326	0.326±0.004	52.910
7.	3.90625	0.425	0.428	0.427	0.427	0.427±0.002	38.432
y=2.4794x+30.664; IC_{50} = 7.79±0.65 (μg/mL)							

Table S16. Average percentage ABTS free radical-scavenging activity of standard reference (Ascorbic acid)

S. N.	Conc. (μg/mL)	R1	R2	R3	Average of R1, R2, R3	Average ± S.D.	% Scavenging activity
1.	20	0.044	0.05	0.049	0.048	0.048±0.003	93.082
2.	10	0.057	0.081	0.09	0.076	0.076±0.017	88.970
3.	5	0.2	0.203	0.221	0.208	0.208±0.011	69.811
4.	2.5	0.292	0.271	0.291	0.285	0.285±0.012	58.684
5.	1.25	0.421	0.422	0.425	0.423	0.423±0.002	38.655
y=5.1916x+39.694; IC_{50} = 1.98±1.20 (μg/mL)							

Table S17: Agglomerative hierarchical cluster (AHC) analysis based on the concentrations of chemical constituents of three *Ocimum* essential oil.

Chemical Components	S-1	S-2	S-3	S-4	S-5	S-6
(3Z)-Hexenol(cis)	0.07	0.07	0.11	0.27	0	0
α-Pinene	0.02	0.12	0.35	0.19	0.19	0.06
Camphene	0	0.06	1.44	0.72	0	0
Benzaldehyde	0	0	0	0.03	0.01	0.05
Sabinene	0.03	0.02	0.09	0.14	0.03	0.03
β-Pinene	0.08	0.05	0.38	0.24	0.2	0.04
6-Methyl-5-hepten-2-one	0	0	0	0	0.04	0.01
1-Octen-3-ol	0.02	0.02	0.1	0.07	0	0
Myrcene	0.01	0	0.58	0.37	0.04	0.04
n-Octanal	0	0	0	0	0.11	0.16
3-octanol	0	0.01	0.15	0.06	0	0
Hexenyl acetate	0	0	0.1	0	0.03	0.03
p-Cymene	0	0	0.18	0.69	0	0
Limonene	0.04	0.06	4.4	3.96	0.05	0.05
1,8-Cineole	0.01	0.06	0.31	0.31	0.05	0.6
(*Z*)-ß-Ocimene(cis)	0.01	0	0.12	0	0.02	0.02
(*E*)-ß-Ocimene(trans)	0.01	0	2.43	0.28	0.21	0.04
Dihydrotagetone	0.02	0	0.15	0.09	0.12	0.03
α-Terpinene	0	0	0.25	0	0	0
cis-Sabinene hydrate	0	0	1.2	1.3	0	0
trans-Linalool oxide	0	0	0	0	0.13	0.18
cis-linalool oxide	0	0	0	0	0.13	0.14
α-Terpinolene	0	0	0.88	0.03	0	0
Linalool	0.02	0.02	9.91	9.72	26.92	27.05
n-Nonanal	0.02	0	0.06	0	0	0
cis-Thujone	0.03	0	0	0	0	0
1-Octen-3-yl acetate	0	0	0	0	0.01	0
α-Campholenal	0	0	0.18	0	0	0
Pinocarvone	0.04	0	0.16	0	0	0
Methyl salicylate	0	0	0.07	0.06	0	0
Borneol	0	0.21	0.46	0.26	0	0
para-1,8-Menthadien-4-ol	0	0	0.1	0.13	0	0
Camphor	0	0	51.03	65.88	0	0
trans-β-Terpineol	0	0	0	0.41	0	0
Terpinen-4-ol	0	0	1.5	1.64	0	0
4-Methyl acetophenone	0	0	0	0.06	0	0
para-Cymen-8-ol	0	0	0.06	0.28	0	0
α-Terpineol	0	0	2.54	1.6	0.01	0
Myrtenol	0	0	0	0.73	0	0
Methylchavicol(Estragole)	0.01	0	0.13	0	64.42	62.16
epi-Borneol	0	0	0.18	0.08	0	0
Neral	0	0	0	0	0.02	0.28
Chavicol	0	0	0	0	0.02	0.01

para-Anis aldehyde	0	0	0	0	0.02	0.02
Geranial	0	0	0	0	0.31	0.11
trans-Anethole	0	0	0	0	0.12	0.16
2-Methyl-2-(para-tolyl) propionaldehyde	0	0	0	0.18	0	0
cis-β-elemene	1.72	1.92	0	0	0	0
trans- β-elemene	29.08	32.85	0	0	0	0
Eugenol	32.15	34.95	0.21	0	0.02	0.02
α-Copaene	0	0	0.56	0.29	0.08	0.08
Methyl eugenol	1.21	1.43	0	0	0.02	0.11
α-Gurjunene	0	0	0	0	0.02	0.02
α-Barbatene	0.19	0.2	0	0	0	0
3-cis-Hexenyl-(3-cis)-hexenoate	0	0	0	0	0.15	0.04
α-Bourbonene	0	0	0.2	0.13	0	0
β-Cubebene	0	0	0.16	0.13	0.05	0.09
β-elemene	0	0	0.38	0.19	0.03	0.07
β-Caryophyllene	19.85	21.64	6.35	3.97	0.79	0.24
Isobazzanene	0.08	0.08	0	0	0	0
β-Copaene	0	0	0.11	0.03	0	0
trans-α-Bergamotene	0	0	0	0	0.58	0.68
cis-β-Farnesene	0	0	0	0	0.03	0.12
trans-β-Farnesene	0	0.06	0	0	0.25	0.14
β-Barbatene	0.22	0.23	0	0	0	0
Dehydro sesquicineole	0.02	0	0	0	0	0
α-Humulene	1.12	1.18	0.37	0.27	0.13	0.44
Germacrene D	0	0	7.75	1.38	0.79	0.14
trans-β-Bergamotene	0	0	0	0	0.1	0
Isobicyclogeramcrene	0	0	1.6	0.3	0	0
Bicyclogermacrene	0	0	0.59	0.04	0.09	0.03
β-Bisabolene	0	0	0	0	0.1	0.02
β-Chamigrene	0.24	0.2	0	0	0	0
β-Selinene	1.06	0.81	0	0	0	0
Valencene	0.04	0.04	0	0	0	0
α-Muurolene	0	0	0.08	0	0	0
α-Selinene	1.01	0.92	0	0	0	0
Germacrene A	1.19	0.69	0	0	0	0
γ-Cadinene	0.07	0	0.05	0	0	0
trans-γ-Bisabolene	0.04	0.07	0	0	2.38	1.84
trans-para-Methoxy cinnamaldehyde	0	0	0	0	0.05	0.02
δ-Cadinene	0	0.01	0.34	0.05	0.04	0.04
Isocaryphyllene oxide	0	0	0	0.09	0	0
Germacrene D-4-ol	0	0	0	0	0.04	0.02
3-cis-Hexenyl benzoate	0	0	0.05	0	0	0
trans-Nerolidol	0	0	0	0	0.07	0.07
Spathulenol	0	0	0	0.29	0	0
10-epi-Cubenol	0.07	0.02	0	0	0	0

Caryophyllene oxide	3.37	0.75	0.24	1.91	0.22	0.64
cis-Thujopsenic acid	0	0	0	0.1	0	0
Humulene epoxide II	0.35	0.03	0	0.07	0	0
neo-Intermedeol	0	0.19	0	0	0	0
β-Eudesmol	0	0	0	0	0.02	0.21
α-Bisabolol	0	0	0	0	0.06	0.12
Intermedeol Isomer	0.15	0.04	0	0	0	0
Caryophyla-4(12),8(13)-dien-5beta-ol	0.14	0	0	0	0	0
iso-Spathulenol	0.97	0.06	0	0	0	0
Neophytadiene	0	0.1	0	0	0	0
Phytadiene isomer	0	0.03	0	0	0	0
epi-α-Cadinol	0	0	0.11	0	0	0
epi-α-Muurolol	0	0	0.08	0	0	0
α-Cadinol	0	0	0.1	0.06	0	0
Dihydro germacrene D	0	0	0	0.12	0	0
allo-Aromadendrene epoxide	0.05	0	0	0.16	0	0
Selin-11-en-4-alpha-ol	1.45	0	0	0	0	0
epi-alpha-Bisabolol	0.04	0	0	0	0	0
Sesquiterpineol	0.63	0	0	0	0	0
Sesquiterpinyl alcohol	1.16	0	0	0	0	0
Tricosane	0	0.03	0	0.05	0	0
Tetracosane	0	0.04	0	0.07	0	0
Pentacosane	0	0.05	0	0.08	0	0
Hexacosane	0	0.04	0	0.07	0	0
Carbonic acid, decyl tridecyl ester	0	0.07	0	0	0	0
Heptacosane	0	0.03	0	0.06	0	0

Note: S-1= O. tenuiflorum, Bardiya-winter; S-2= O. tenuiflorum, Bardiya-autumn; S-3= O. americanum, Thankot-winter; S-4= O. americanum, Thankot-summer; S-5 = O. basilicum, Kapilvastu-winter; S-6= O. basilicum, Kapilvastu-summer.
